# ER stress genes (*COL1A1, LOXL2, VWF*) predicts IKK-16 as a Candidate therapeutic target for colitis-related inflammation and fibrosis suppression

**DOI:** 10.3389/fimmu.2025.1587860

**Published:** 2025-06-18

**Authors:** Ke Zhang, Jiao Yang, Qing-Qing Yang, Jun-An Guo, Qian-Hui Huang, Chan Cui, Yue-Mei Wang, Qiao-Feng Wu, Jun-Meng Wang

**Affiliations:** ^1^ Acupuncture and Tuina College, Chengdu University of Traditional Chinese Medicine, Chengdu, Sichuan, China; ^2^ Key Laboratory of Acupuncture for Senile Disease, Chengdu University of Traditional Chinese Medicine, Ministry of Education, Chengdu, China

**Keywords:** intestinal fibrosis, inflammation, ER stress, molecular docking, bioinformatics analysis, immune infiltration

## Abstract

**Introduction:**

The role of endoplasmic reticulum stress (ERS) in the immune-inflammatory dysregulation and intestinal fibrosis associated with ulcerative colitis (UC) remains unclear. This study aims to identify ERS-related genes involved in UC fibrosis and explore potential therapeutic targets.

**Methods:**

Differentially expressed ERS-related genes (DE-ERGs) were identified through comprehensive analysis of public datasets. Machine learning methods screened VWF, MZB1, COL1A1, and LOXL2 as key regulators. Immune infiltration analysis, protein-protein interaction (PPI) network analysis, and gene set variation analysis (GSVA) were performed to clarify their roles in UC fibrosis. Drug prediction was conducted using the Connectivity Map (CMap) database, supplemented by a literature review.

**Results:**

The predicted drugs were ranked based on their binding affinities as follows: IKK-16 > Quercetin > Curcumin > Resveratrol > Budesonide > Trimebutine > Colchicine > Betamethasone > Pioglitazone > Metformin. IKK-16 showed the highest binding affinity for treating UC fibrosis. COL1A1, LOXL2, and VWF were identified as key drivers of UC intestinal fibrosis, supported by immune infiltration and PPI network analyses.

**Discussion:**

These results suggest that ERS-related genes, particularly COL1A1, LOXL2, and VWF, may regulate UC fibrosis through interactions with immune cells. IKK-16 shows promise as a therapeutic agent. These findings provide new insights into UC pathogenesis and potential clinical treatment strategies.

## 1 Introduction

Ulcerative colitis (UC), a major subtype of inflammatory bowel disease (IBD), is an immune-mediated chronic inflammatory disorder driven by dysregulated interactions between innate and adaptive immunity. Globally, the annual incidence of UC is on the rise, with the fastest increases observed in countries across Asia, Africa, and South America (1). Currently, in East Asia, UC affects approximately 4.59 to 57.3 individuals per 100,000 people, making it a global public health challenge (2). The clinical symptoms of UC include hematochezia, diarrhea, abdominal pain, and tenesmus. Its pathological features are hyperactive immune responses and destruction of the colorectal epithelium, with complex pathological factors that severely affect patients’ daily life and work. In recent years, with the deepening of research, it has been found that intestinal fibrosis may be an important reason for the chronicity and refractoriness of UC (3, 4), but the underlying mechanisms remain unclear.

Endoplasmic reticulum stress (ERS) arises from disrupted ER homeostasis due to pathogenic stimuli, triggering cellular adaptive responses to misfolded/unfolded protein accumulation (5–7). Emerging evidence highlights ERS as a critical driver of fibrotic pathogenesis across multiple organs. In pulmonary fibrosis, ERS-mediated activation of the unfolded protein response (UPR) and subsequent apoptosis in alveolar epithelial cells have been mechanistically linked to fibrogenesis (8, 9) with additional contributions from ECM overproduction and inflammatory cascade amplification. Hepatic fibrosis studies reveal ERS-induced activation of hepatic stellate cells (HSCs) driving pathological ECM deposition (10, 11), while similar mechanisms underlie renal (12, 13) and cardiac (14, 15) fibrotic remodeling. The pro-fibrotic mechanisms of ERS converge on three principal pathways (16, 17): UPR-mediated proteostasis regulation through IRE1, PERK, and ATF6 signaling branches, where sustained activation shifts from adaptive to apoptotic responses; Fibroblast activation and pathological ECM synthesis; Inflammatory pathway potentiation through crosstalk with NF-κB and NLRP3 inflammasomes. It is evident that ERS may be widely involved in tissue fibrosis, and its significant importance requires further research.

Is there a relationship between endoplasmic reticulum stress and intestinal fibrosis? Grootjans J and Xie M have reported that endoplasmic reticulum stress can lead to intestinal fibrosis by activating apoptosis and fibrosis-promoting signaling pathways in intestinal epithelial cells and fibroblasts (18, 19). However, their studies observed the occurrence of endoplasmic reticulum stress and the regulatory factors of gut fibrosis. As a typical inflammatory disease, is there a correlation between immune-inflammatory dysregulation, ERS and intestinal fibrosis? How do they interact or co-act? Are there potential therapeutic targets that can improve immune inflammation while also inhibiting intestinal fibrosis and ERS? In this study, we conducted a comprehensive analysis of existing bioinformatics data on ulcerative colitis, first screening key genes mainly related to immune-inflammatory dysregulation, and further obtained the key genes involved in ERS by intersecting with the ERS dataset. Then, we analyze their relationship with intestinal fibrosis. The results show that, as a typical immune-inflammatory dysregulation disease, some of the key genes that change significantly in UC are indeed closely related to ERS and intestinal fibrosis. By constructing a PPI network and performing GSVA, we validated the potential key roles of these genes in fibrosis regulation. In addition, we have used small molecule docking combined with network pharmacology analysis to predict potential drugs for the clinical treatment of UC intestinal fibrosis and speculated on potential traditional Chinese medicines that may treat ulcerative colitis based on the predicted compounds, finding consistency with clinical practices, which indirectly verifies the role of this target in UC intestinal fibrosis.

## 2 Results

### 2.1 KEGG analysis reveals significant enrichment of inflammatory and immune response pathways in ulcerative colitis

**Figure 1:**
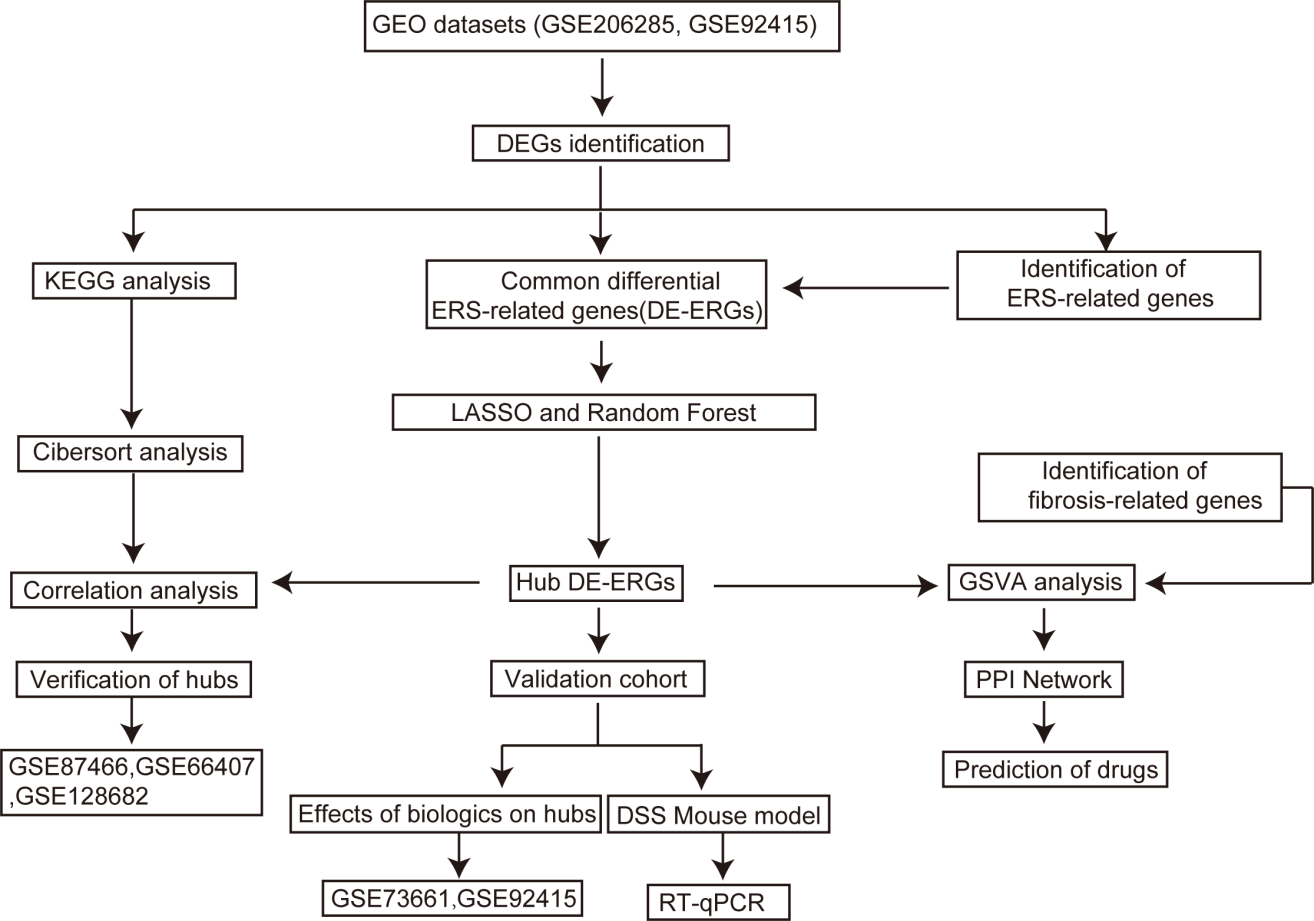
Flowchart of the study.

**Figure 2:**
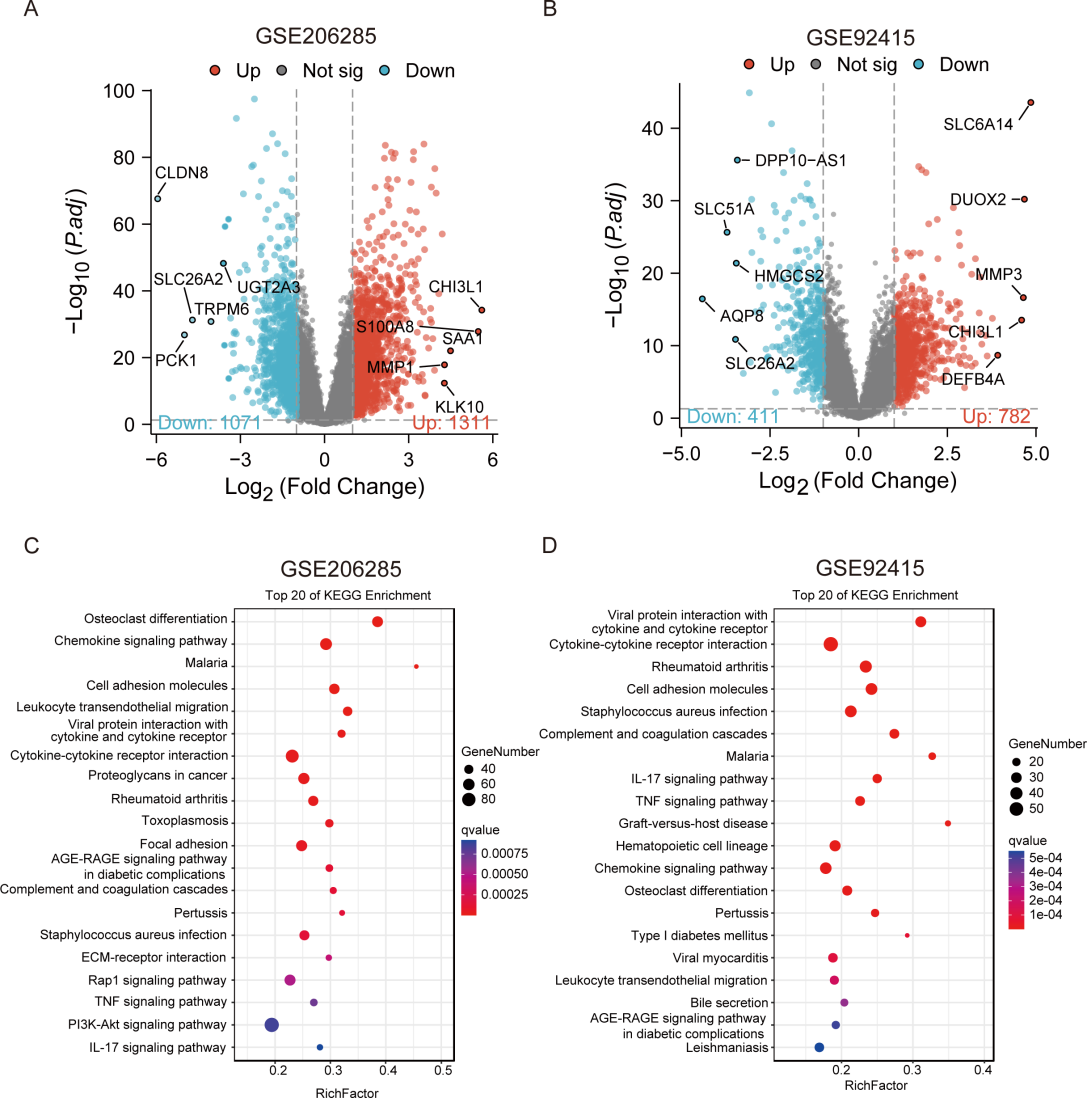
The identification and functional enrichment analysis of DEGs between the UC group and CON group in two training datasets. **(A, B)** Volcano plot representation of differential gene expression in GSE206285 and GSE92415. **(C, D)** KEGG pathway enrichment analysis of DEGs in GSE206285 and GSE92415.

The study workflow is presented in **Figure 1**. The sets GSE206285 (20) and GSE92415 (21) were normalized, and the gene expression with biological significance was obtained. In set GSE206285, there were 2382 genes differentially expressed between 550 UC samples and 18 healthy control samples, including 1311 upregulated genes and 1071 downregulated genes (**Figure 2A**). For another independent set GSE92415, which contained 162 UC samples and 21 healthy control samples, there were 782 upregulated genes and 411 downregulated genes identified (**Figure 2B**). The common DEGs of GSE206285 and GSE92415 include downregulated *aqp8*, hmgcs2, and upregulated *chi3l1, mmp* subfamily gene like *mmp1* and *mmp3* (**Supplementary Table 3**). In addition, to clarify the biological characteristics of these DEGs, we performed KEGG enrichment analysis on two datasets separately to understand which signaling pathways may play important roles in UC disease. The results showed that among the top 20 enriched pathways in both datasets, various inflammation-related pathways were enriched, including “*Chemokine signaling pathway*”, “*cytokine-cytokine receptor*”, “*IL-17 signaling pathway*”, “*tumor necrosis factor (TNF) signaling pathway*”, “*complement and coagulation cascades*” (**Figures 2C, D**). These results indicate that pathways associated with inflammation and immune response are prominently enriched in UC patients.

### 2.2 VWF, MZB1, COL1A1, and LOXL2 are key genes involved in endoplasmic reticulum stress

**Figure 3:**
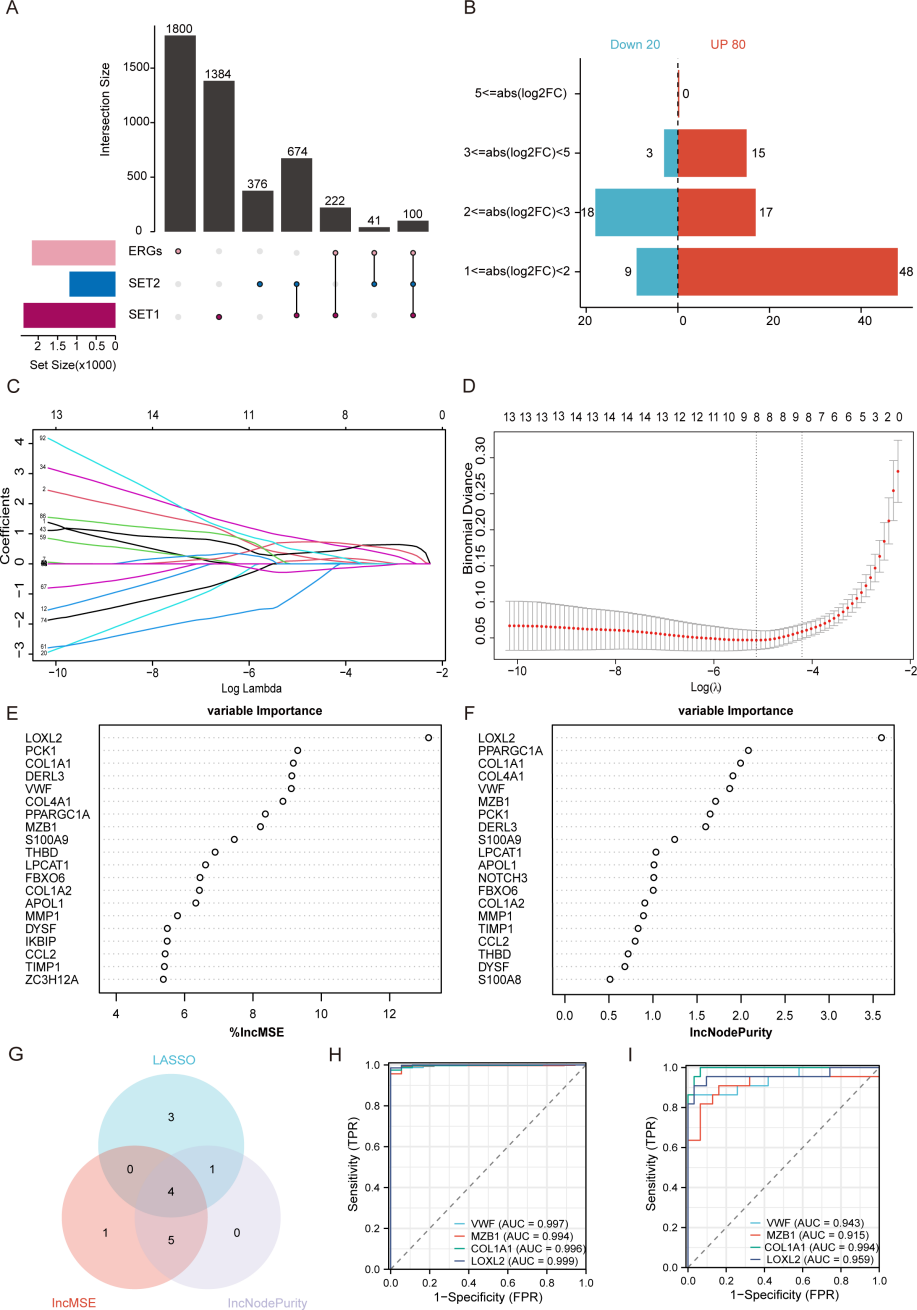
Selection and identification of Hub DE-ERGs using machine learning methods. **(A)** The upset plot shows the overlap between ERGs and DEGs of the two training sets. **(B)** The bidirectional bar plot illustrates the expression trends of 100 DE-ERGs under different LogFC conditions consistent with two identical training sets. The logFC values indicated in the figure (logFC = 1, 2, 3, 5) correspond to fold changes of 2-fold, 4-fold, 8-fold, and 32-fold in expression levels, respectively. **(C)** LASSO coefficient profiles of the 100 DE-DEGs. Different colors are used for visual differentiation between genes. **(D)** Cross-validation selects the optimal tuning parameter log(λ) in LASSO regression analysis, filtering out 8 most strongly correlated DE-DEGs. **(E)** The Increment mean squared error method (IncMSE) and **(F)** gini coefficient method (IncNodePurity) in a random forest classifier yielded the following results. The importance index is on the x-axis, and the genetic variable is on the y-axis. **(G)** The intersection of LASSO, IncMSE, and IncNodePurity results. **(H)** ROC curves of hub-genes in the GSE206285 dataset. **(I)** ROC curves of hub-genes in the GSE92415 dataset.

Endoplasmic reticulum stress-related genes (ERGs) were obtained from the Gene Cards database, and these genes were overlapped with DEGs screened from the SET1(GSE206285) and SET2(GSE92415) datasets, resulting in 100 overlapping ER stress-related differentially expressed genes (DE-ERGs) (**Figure 3A**). To validate the reliability of DE-ERGs, we screened them under different LogFC criteria. The results showed that among the 100 DE-ERGs, 80 genes were upregulated and 20 genes were downregulated. (**Figure 3B**). To further improve the quality of key DE-ERGs of UC, we used two different machine learning algorithms to screen genes. We performed variable selection and model simplification using the regularization method in LASSO regression, and applied cross-validation to achieve optimal model performance and avoid overfitting. As a result, 8 feature DE-ERGs were identified (**Figures 3C, D**). Additionally, we determined the top 20 feature genes using Random Forest analysis based on the Incremental Mean Squared Error and Gini coefficient methods (**Figures 3E, F**). Combining the results of the above three algorithms, the top 10 genes ranked by random forest analysis were selected, and a Venn diagram is used to identify four intersecting DE-ERGs (**Figure 3G**). Subsequently, we performed ROC analysis to verify the diagnostic performance of the four intersecting DE-ERGs (**Figures 3H, I**). The area under the curve (AUC) value > 0.90 for all genes, indicating that they exhibit excellent responsiveness to the prediction of UC, Finally, *VWF, MZB1, COL1A1 and LOXL2* were determined as hub genes.

### 2.3 Immune cell correlation analysis and validation reveal that VWF, MZB1, COL1A1 and LOXL2 are positively correlated with UC inflammation

**Figure 4:**
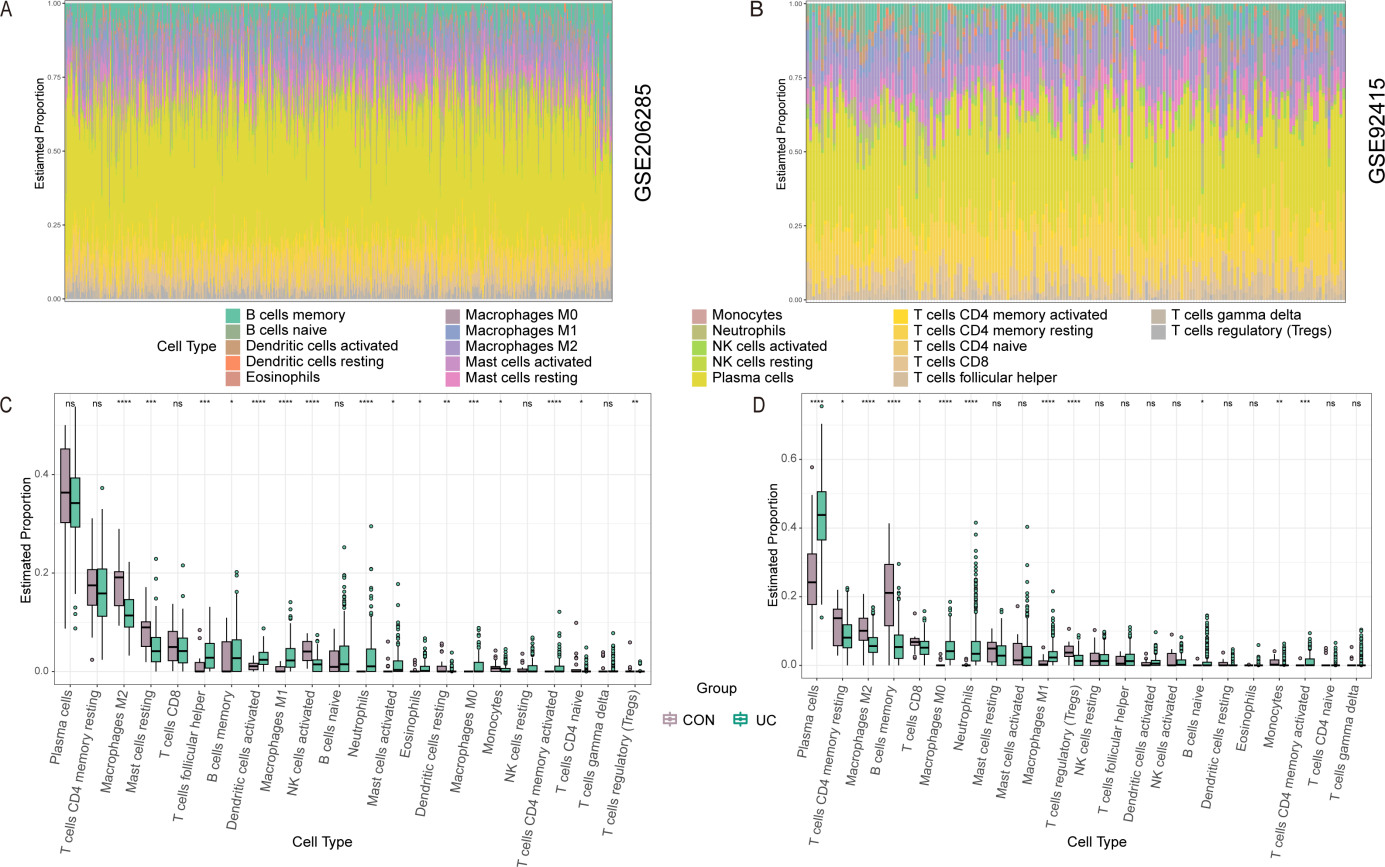
The immune characteristics between the UC group and healthy controls in the GSE206285 and GSE92415 via CIBERSORT. **(A, B)** Stacked bar graph show the relative composition of 22 immune cell subsets in the two datasets. **(C, D)** Boxplots show that the difference in the proportion of immune cells between the CON and UC groups in the two datasets. Data were assessed via the method of Benjamini and Hochberg (BH). * adj. p-value < 0.05, ** adj. p-value < 0.01, *** adj. p-value < 0.001, **** adj. p-value < 0.0001, ns, no significance.

**Figure 5:**
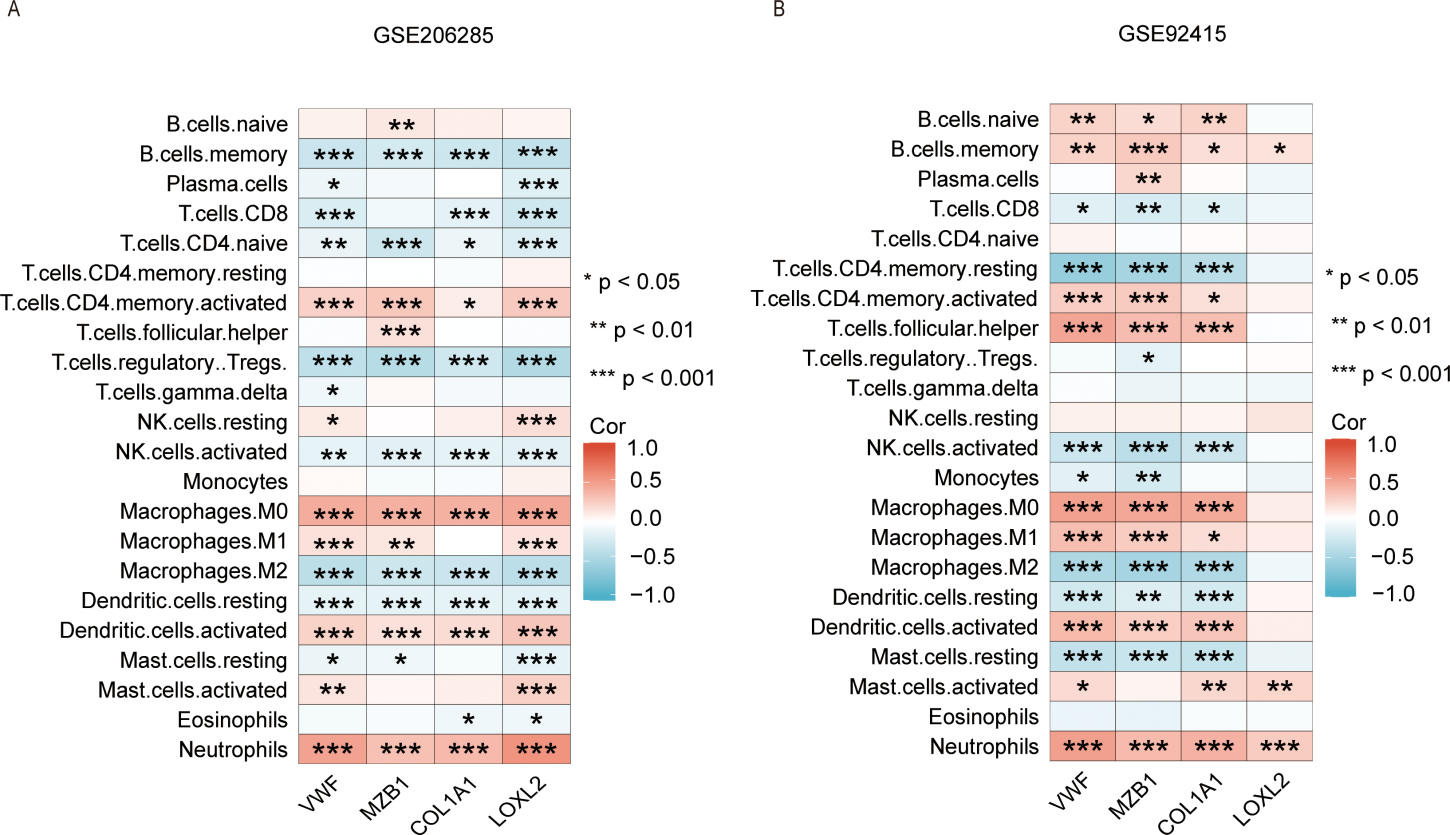
Correlation analysis of hubs with immune cells in the GSE206285 and GSE92415. **(A, B)** Heatmap delineating the correlation hubs with 22 immune cell types. The color scale represents Pearson correlation coefficients (Cor) ranging from –1 to 1, with red indicating positive correlation and blue indicating negative correlation. Asterisks denote statistical significance. P-values were calculated using Pearson correlation analysis and adjusted for multiple testing using the Benjamini-Hochberg method (FDR). *P < 0.05, **P< 0.01, ***P < 0.001.

Ulcerative colitis (UC) is an autoimmune disease characterized by aberrant immune system activity playing a crucial role in its pathogenesis. Previous research has indicated that differentially expressed genes between two datasets are predominantly enriched in pathways associated with inflammatory responses. To better understand the immune cell characteristics in UC colonic tissue, we utilized the CIBERSORT algorithm to delineate the abundance of 22 immune cell types across both groups of colon tissues. **Figures 4A, B** shows the distribution of immune cell types in each sample in the two training sets. This suggests that these immune cells may play important roles in the pathogenesis of the disease, laying a foundation for further functional studies. In contrast, **Figures 4C, D** illustrates the difference in the abundance of infiltrated immune cells between the two groups. The results revealed that the UC tissue was infiltrated by a higher fraction of macrophages (M0 and M1), Neutrophils and activated CD4 memory T cells and a lower fraction of M2 and Tregs. Next, the correlation between the four hub genes and immune cells was examined. Similar to the above results, macrophages (M0 and M1), Neutrophils, and activated CD4 memory T cells were positively correlated with VWF, MZB1, COL1A1, and LOXL2 (**Figures 5A, B**). Further supports the potential role of these hub genes in the immunopathogenesis of UC.

**Figure 6:**
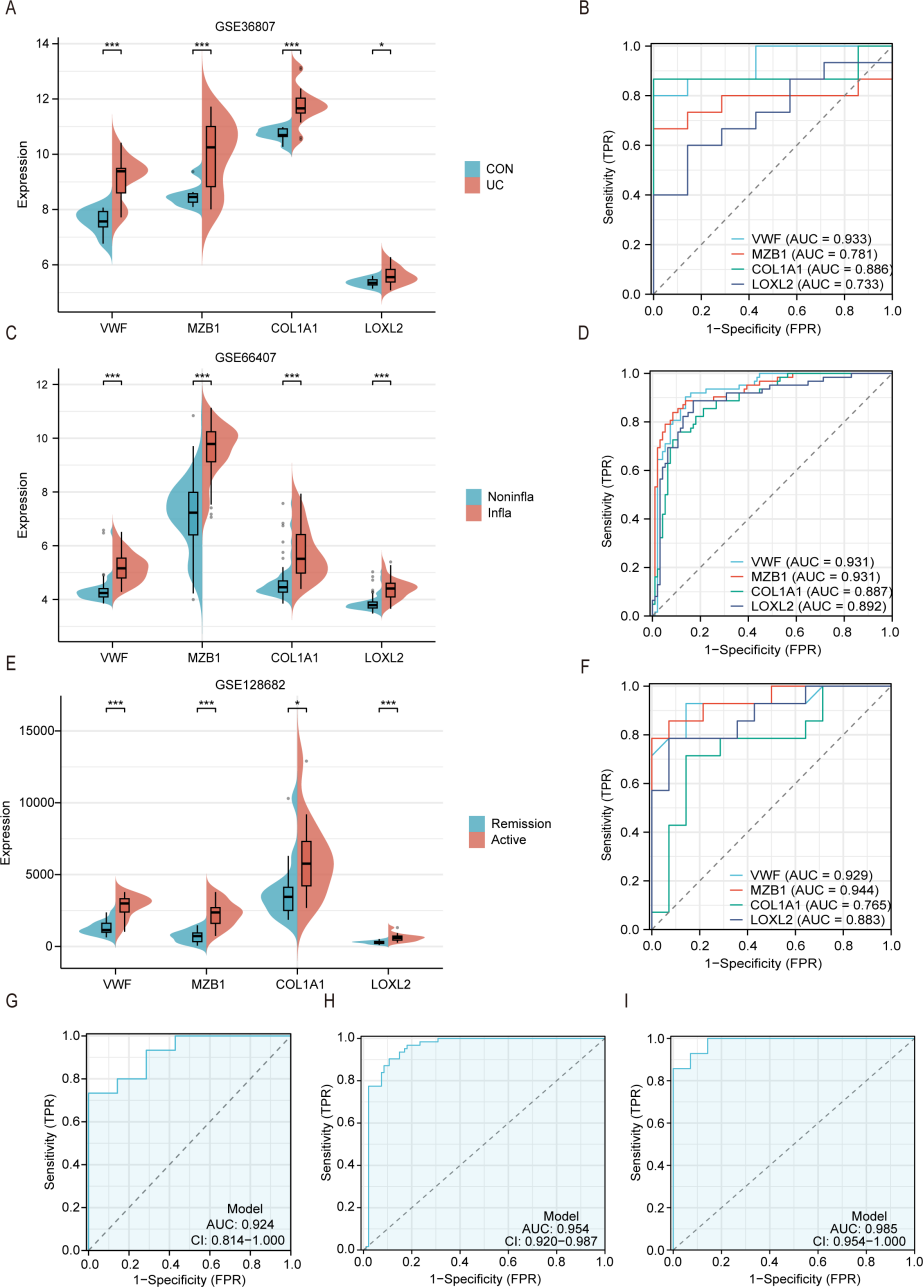
External datasets validated the high expression of hub genes in patients with active UC and colonic inflammatory tissues. **(A)** Split violin plot revealing the expression differences in hubs between UC patients and healthy controls in the GSE36807. **(B)** ROC curves of hubs in the GSE36807. **(C)** Split violin plot revealing the expression differences in hubs between inflammatory tissues and non-inflammatory tissues in the GSE66407. **(D)** ROC curves of hubs in the GSE66407. **(E)** Split violin plot revealing the expression differences in hubs active UC and remission patients in the GSE128682. **(F)** ROC curves of hubs in the GSE128682. **(G-I)** ROC analysis based on hubs combination in GSE36807 **(G)**, GSE66407 **(H)** and GSE128682 **(I)**. *P < 0.05, ***P < 0.001.

To validate the expression levels of hub-genes, we used three external validation datasets (GSE36807, GSE66407, GSE128682) (22, 23). As shown in the figures, the expression levels of hub-genes in colonic tissues of UC patients were significantly higher than those in healthy controls (**Figure 6A**). ROC analysis demonstrated that the AUCs of all hub-genes were >0.70 (**Figure 6B**). Furthermore, differential expression analysis between inflamed and non-inflamed tissues in GSE66407 revealed that hub genes positively correlated with immune cells showed higher expression levels in inflamed tissues (**Figure 6C**). The AUCs of the four hub-genes were all >0.85 (**Figure 6D**). Subsequently, we analyzed the levels of these genes between active UC and remission patients in GSE128682. All hubs were upregulated in the diseased colonic tissues of active UC patients (**Figure 6E**), with AUCs >0.75 (**Figure 6F**). These results indicate that these four hubs have good diagnostic performance in assessing UC disease. Finally, we performed ROC analysis using a multi-gene combination to evaluate the predictive ability of three models, demonstrating that the AUCs of all three datasets were >0.90 (**Figures 6G-I**), confirming excellent evaluation efficacy of these three models.

### 2.4 Clinical sample response to biologics and animal experiments confirm the important roles of hub genes in the pathogenesis of UC

**Figure 7:**
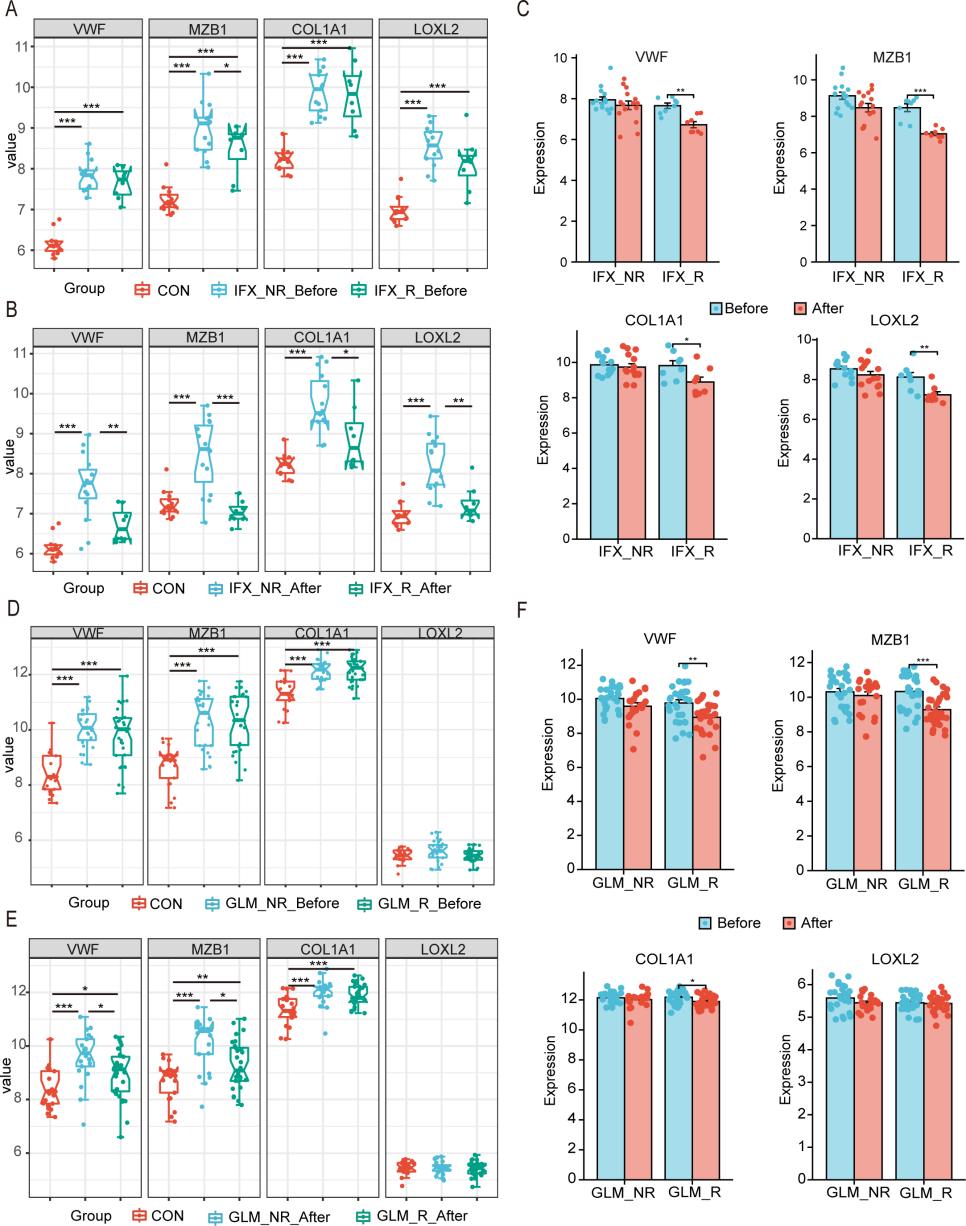
Evaluate the impact of biologics on hub genes using drug sensitivity analysis. **(A-C)** The relative expression levels of hubs in the colonic mucosa of healthy controls, UC patients in not responding and responding groups before and after IFX therapy. **(D-F)** The relative expression levels of hubs in the colonic mucosal of healthy controls, UC patients in responding and non-responding groups before and after GLM treatment. IFX, infliximab; GLM, golimumab. *P < 0.05, **P< 0.01, ***P < 0.001.

Clinical research indicates that biologics used for UC treatment are ineffective or minimally effective in some patients. To determine if these hub genes can guide personalized medication, we investigated their correlation with TNF-α inhibitor infliximab (IFX) and the first-line UC treatment, golimumab (GLM), using datasets GSE73661 (24) and GSE92415. we found that compared to non-responders (IFX_NR_Before), responders (IFX_R_Before) exhibited a significant reduction in MZB1 expression (**Figure 7A**). Following IFX treatment, the expression levels of VWF, MZB1, COL1A1 and LOXL2 in responders (IFX_R_After) all returned to those observed in healthy controls (**Figure 7B**). Notably, hub-genes expression was significantly reduced post-IFX treatment compared to pre-treatment levels (**Figure 7C**). Further analysis of GSE92415, revealed that before treatment, active UC patients showed increased expression of VWF, MZB1, and COL1A1, while LOXL2 no response compared to the control group (**Figure 7D**). However, after GLM treatment, none of the four hub genes returned to healthy control levels (**Figure 7E**). Additionally, compared to pre-treatment, the clinical response group showed significant reductions in the expression of the other three hub genes, except for LOXL2, which remained unresponsive. (**Figure 7F**). These findings indicate that IFX and GLM modulate endoplasmic reticulum stress-related genes in patients responding to treatment.

**Figure 8:**
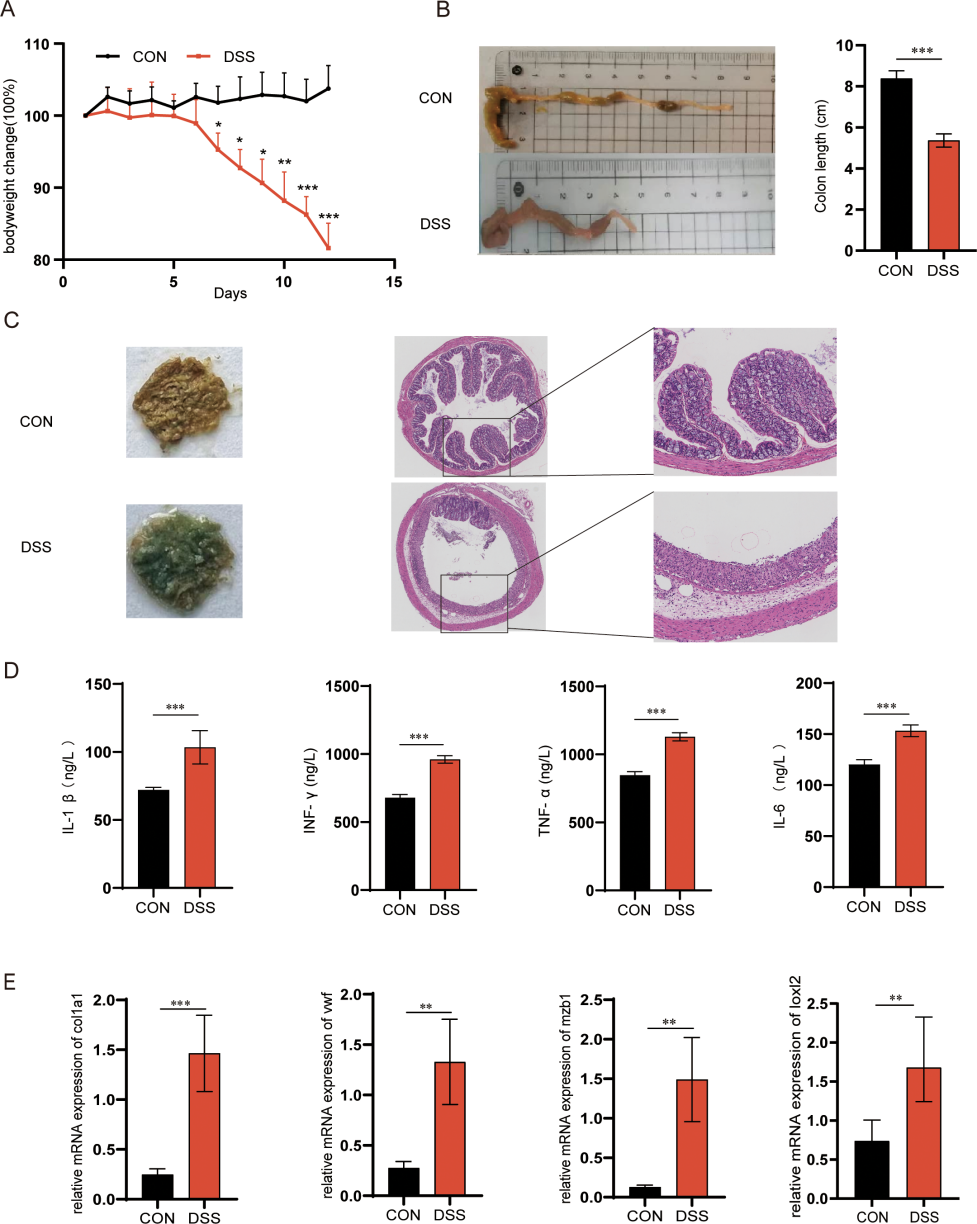
Animal models validated the hubs related to endoplasmic reticulum stress. **(A)** Body weight changes (n=6), **(B)** Colonic length changes (n=6) between CON and UC mice. **(C)** mice with bloody stools and Representative H&E staining of the colon (magnification ×100, n=6), **(D)** The expression of TNF-α, INF-γ, IL-1β and IL-6 in the serum. **(E)** The mRNA expression levels of the hub genes, Vwf, Mzb1, Loxl2 and Col1a1 in UC and CON samples by RT-qPCR (n=6). Statistical analysis by two-sided Student’s t tests in **(B, D)** INF-γ and IL-6, the Mann-Whitney U test in **(D)** TNF-α, the Welch test in **(D)** IL-1β and **(E)**. *P<0.05, **P<0.01, ***P<0.001. Data are show as mean ± SD.

Next, to further validate the important role of hub genes in active UC, we employed the DSS-induced mouse colitis model. Compared to the CON group, the DSS-induced mice showed increased body weight loss (P < 0.05、P < 0.01 and P < 0.001, **Figure 8A**), positive fecal occult blood, incomplete colon morphology and shortened length (P < 0.001, **Figures 8B, C**), as well as increased serum inflammatory factors TNF-α, INF-γ, IL-1β, and IL-6 (P < 0.001, **Figure 8D**). Furthermore, we examined the mRNA expression levels of VWF, MZB1, COL1A1 and LOXL2 in the colon tissues of UC with RT-qPCR to verify the reliability of the results. Consistent with previous results, the expression levels of Vwf, Mzb1, Col1a1 and Loxl2 in the DSS group were significantly higher than those in the CON group (P < 0.01 and P < 0.001, **Figure 8E**), supporting a potential association between these ER stress-related genes and UC pathogenesis. While the results suggest their possible involvement in disease progression, further functional studies such as gene knockdown or knockout experiments are warranted to clarify their regulatory roles.

### 2.5 PPI network and GSVA analysis demonstrated the interaction of COL1A1, LOXL2, and VWF in the process of intestinal fibrosis in UC

**Figure 9:**
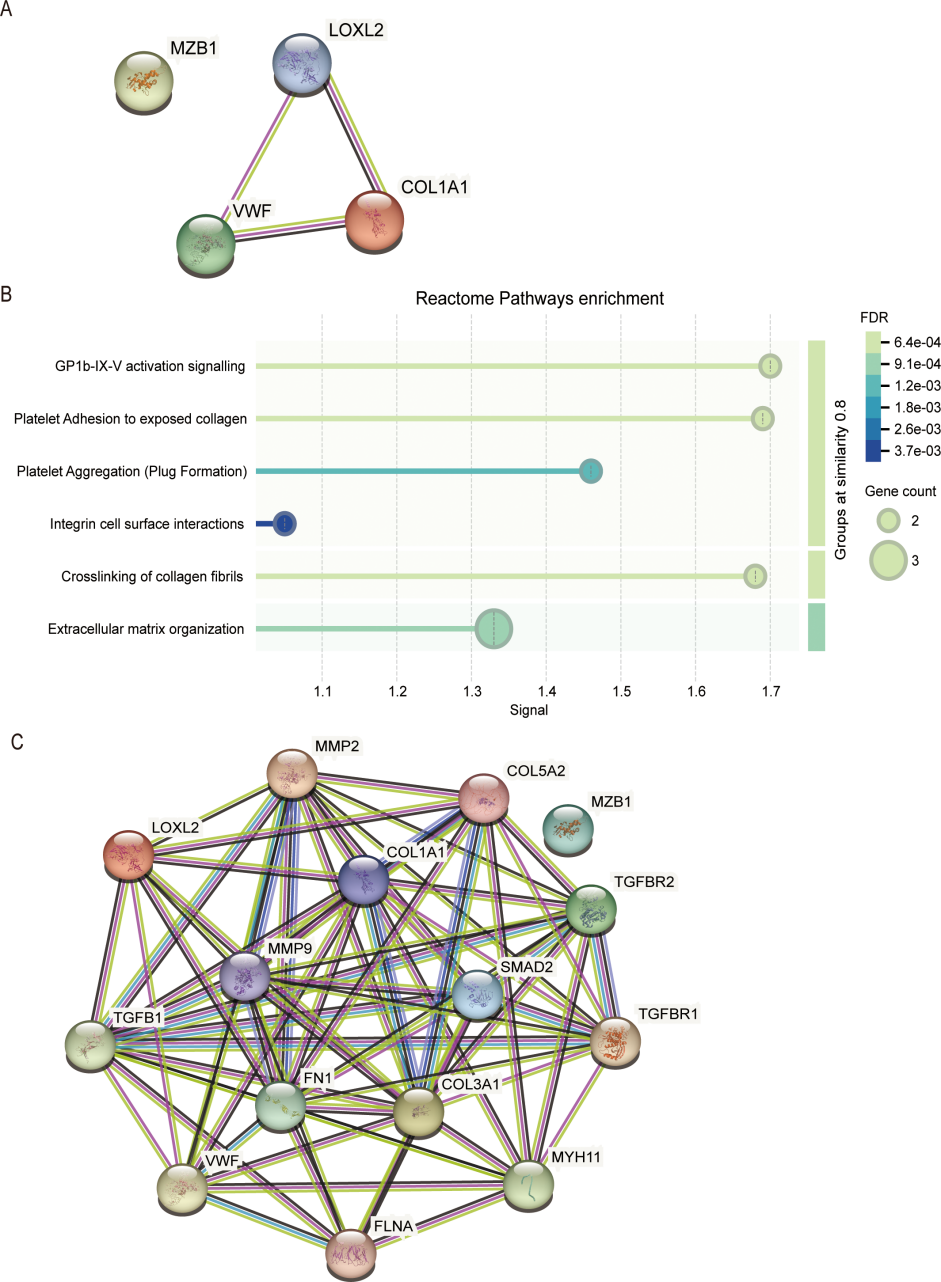
PPI network construction and pathway enrichment analysis of fibrosis-related genes. **(A)** Visualization of the PPI network of hub genes (COL1A1, LOXL2, VWF, and MZB1) using STRING. **(B)** Reactome pathway enrichment analysis of hub genes revealed major pathways associated with extracellular matrix organization and collagen fibril crosslinking. The size of the dots represents the number of genes in each pathway, while the color indicates the FDR value. The signal strength on the x-axis reflects the significance of the pathways. **(C)** The extended PPI network illustrates the interactions between hub genes and classical fibrosis-related genes. Nodes represent proteins, and the edge colors indicate the type of interaction (e.g., experimental evidence, co-expression).

To clarify the key mechanism of action of the hub genes, we constructed a protein-protein interaction (PPI) network using the STRING database. The results showed that, except for MZB1, COL1A1, LOXL2, and VWF interact with each other (**Figure 9A**). Reactome Pathway analysis indicated that these genes are primarily enriched in the *‘GP1b-IX-V activation signaling,’ ‘Platelet Adhesion to exposed collagen,’* and *‘Crosslinking of collagen fibrils’* pathways (**Figure 9B**). Further analysis indicates that these three genes, along with upstream and downstream factors of classical fibrosis pathways, can form a PPI network. As shown in the figure, except for MZB1, COL1A1, LOXL2, and VWF interact with these fibrosis-related genes such as MMP9, FN1, COL3A1, and TGFB1 (**Figure 9C**). Gene Ontology (GO) Biological Process enrichment analysis revealed that these genes are primarily enriched in the *‘Positive regulation of epithelial-to-mesenchymal transition’* and *‘Extracellular matrix organization,’* suggesting that these genes may be involved in cell migration, tissue remodeling, and fibrosis (S1A). WikiPathways enrichment analysis identified *‘Canonical and non-canonical TGF-β signaling’* and *‘NRP1-triggered signaling pathways in pancreatic cancer’* as significantly enriched pathways, further indicating that these genes may participate in TGF-β-related signaling regulation, supporting the potential molecular mechanisms of fibrosis (S1B). These results support the role of COL1A1, LOXL2, and VWF in extracellular matrix remodeling and fibrosis.

**Figure 10:**
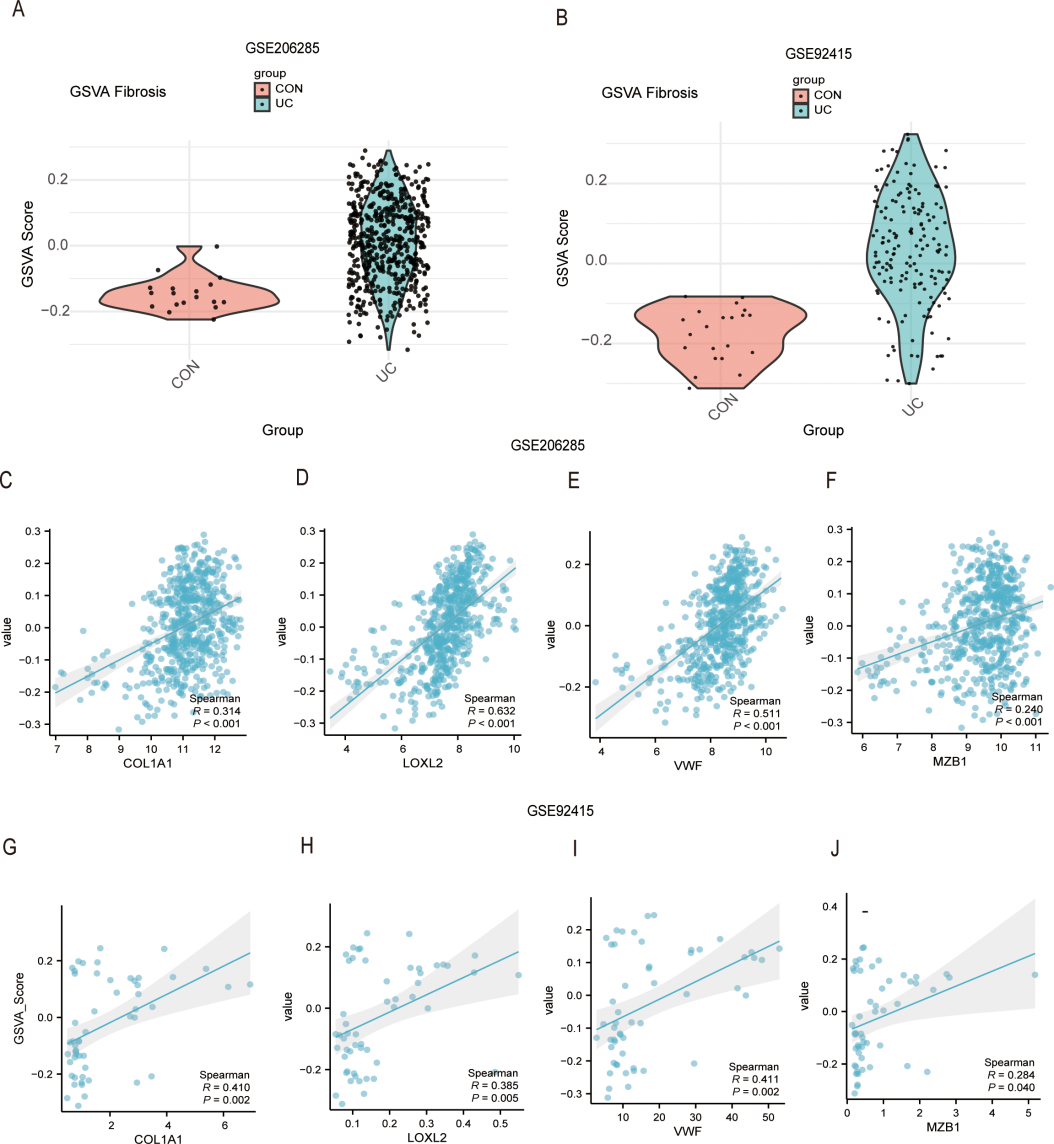
Correlation analysis of hub genes with fibrosis-related GSVA scores in GSE206285 and GSE92415 datasets. **(A, B)** Violin plots showing the distribution of GSVA scores for fibrosis-related gene sets between the CON (control) and UC (ulcerative colitis) groups in the GSE206285 **(A)** and GSE92415 **(B)** datasets. **(C-F)** Scatter plots depicting the correlation between fibrosis-related GSVA scores and the expression levels of COL1A1, LOXL2, VWF, and MZB1 in the GSE206285 dataset. **(G-J)** Scatter plots showing the relationship between hub gene expression levels and GSVA scores in the GSE92415 dataset. The x-axis represents gene expression levels, and the y-axis represents GSVA scores. Each dot corresponds to an individual sample. Sample sizes: GSE206285 - CON (n = 18), UC (n = 550); GSE92415 - CON (n = 21), UC (n = 162). The trend line indicates the direction of linear correlation, and the gray shading indicates the confidence interval for the linear fit. Spearman’s correlation coefficient (R) > 0 indicates a positive correlation, and a p-value < 0.05 denotes statistical significance.

To clarify whether these endoplasmic reticulum stress-related hub genes are involved in the fibrosis process of UC, we obtained a fibrosis-associated gene set (FGS) from the Gene Cards database (as described in the methods section). We then analyzed the activity changes of this gene set in different samples using the GSVA method, combined with the GSE206285 and GSE92415 datasets. The results showed that, compared with the CON group, the GSVA score in the UC group was significantly elevated, indicating that the fibrosis-related gene set is more active in the UC group (**Figures 10A, B**). Due to the smaller sample size in the control group, which may affect the robustness of the results, we applied a non-parametric test to reduce bias. Despite this limitation, a consistent trend of increased GSVA scores was observed in the UC group across both datasets, and this suggests that fibrosis pathways are more easily activated in ulcerative colitis. Next, we further explored the correlation between the expression levels of COL1A1, LOXL2, VWF, and MZB1 genes and the activity of the gene set. In the GSE206285 dataset, the GSVA score of the fibrosis-related gene set showed a significant positive correlation with the expression levels of COL1A1 (Spearman R = 0.314, P < 0.001), LOXL2 (Spearman R = 0.632, P < 0.001), VWF (Spearman R = 0.511, P < 0.001), and MZB1 (Spearman R = 0.240, P < 0.001) (**Figures 10C-F**), suggesting that the expression levels of these genes may be closely related to the activity of fibrosis-related pathways. In the GSE92415 dataset, similar correlations were observed between the fibrosis-related gene set activity score and the expression levels of COL1A1 (Spearman R = 0.410, P = 0.002), LOXL2 (Spearman R = 0.385, P = 0.005), VWF (Spearman R = 0.411, P = 0.002), and MZB1 (Spearman R = 0.284, P = 0.040) (**Figures 10G-J**), further validating their potential key roles in fibrosis regulation. Consistent with the aforementioned PPI results, MZB1 showed the weakest correlation with the fibrosis gene set in both datasets. Through this approach, we demonstrated that COL1A1, LOXL2, and VWF may participate in the fibrotic pathological process of ulcerative colitis by regulating fibrosis-related pathways.

### 2.6 IKK-16 may represent a potential therapeutic agent for UC-associated intestinal fibrosis by targeting ERS

In order to identify potential drugs for treating UC-related intestinal fibrosis, a search through the CMap database and the literature resulted in the identification of 11 potential drugs, including the predicted small molecule compound IKK-16 (**Supplementary Table 1**) and the top 10 antifibrotic drugs (**Table 1**, **Supplementary Table 4**). Among them, the optimized crystal structures of hub genes with a resolution lower than 3 Å were downloaded from the PDB website (S2A-D), and the 3D structures of small molecule compounds were obtained from the PubChem website (S2E-O), with specific IDs listed in **Supplementary Table 2**. Next, to evaluate the binding affinity of these small molecule compounds to target genes, we performed molecular docking analysis using Autodock Vina 1.2.2 to identify the best conformations of drug-protein interactions. Ultimately, we obtained binding poses and interactions for 11 candidate drugs with 4 proteins and ranked the candidates based on binding energy (**Table 2**).

**Table 1:** Drug prediction table.

Generic Name	Chemical Formula	Mechanism of Action	Can it treat fibrosis?	Does it target gastrointestinal diseases?	References
Metformin	C_4_H_11_N_5_	Multidrug and toxin extrusion protein 1 Modulator; Acetyl-CoA carboxylase 2 Activator; Electron transfer flavoprotein-ubiquinone oxidoreductase, mitochondrial Inhibitor	YES (Intestinal Fibrosis)	YES	Wang Y, et al. China.2022 (25)
Budesonide	C_25_H_34_O_6_	Glucocorticoid receptor Agonist	YES (Liver, pulmonary, renal, intestinal, cystic, esophageal fibrosis.)	YES	Artone S, et al. Italy.2024 (26)
Curcumin	C_21_H_20_O_6_	Collagenase 3; Prostaglandin G/H synthase 1; Matrix metalloproteinase-9	YES (Liver, pulmonary, renal, cardiac fibrosis.)	YES	Gupta SC, et al. USA. 2012 (27)
Pirfenidone	C_12_H_11_NO	Transforming growth factor beta-1 proprotein Modulator	YES (pulmonary, Intestinal Fibrosis)	YES	Kim ES, et al. New Zealand. 2015 (28) Cao X, et al. China. 2024 (29) Cui Y, et al. Netherlands. 2020 (30)
Resveratrol	C_14_H_12_O_3_	NAD-dependent protein deacetylase sirtuin-1Inhibitor	YES (Liver, renal, intestinal, cystic fibrosis.)	YES	Li P, et al. China.2014 (14) Garcia P, et al. USA. 2012 (15)
Fraxinellone	C_14_H_16_O_3_	/	YES	YES	Wang J, et al. China, 2023 (31)
Quercetin	C_15_H_10_O_7_	Signal transducer and activator of transcription 3 Inhibitor; Lysophosphatidylcholine acyltransferase 2 Inhibitor; Lysophosphatidylcholine acyltransferase 1 Inhibitor.	YES (pulmonary fibrosis, Wound healing.)	YES	Wu W, et al. China, 2024 (32)
Colchicine	C_22_H_25_NO_6_	Tubulin beta chain Inhibitor Binder	YES (Liver, pulmonary fibrosis.)	/	Leung YY, et al. Singapore. 2015 (33) Kaplan MM, et al. USA. 2010 (34)
Aminocaproic acid	C_6_H_13_NO_2_	Plasminogen Inhibitor; Tissue-type plasminogen activator Antagonist	YES (wound healing)	YES	Wan Z, et al. China, 2022 (35)
Trimebutine	C_22_H_29_NO_5_	Mu-type opioid receptor Agonist; Voltage-dependent L-type calcium channel Inhibitor; Calcium-activated potassium channel Inhibitor.	NO	YES	Lee HT, et al. Korea. 2011 (36)

**Table 2:** Binding energy for targets with their drugs.

Drugs	Binding Energy(kcal/mol)			
	COL1A1	LOXL2	VWF	MZB1
IKK-16	-8.241	-8.93	-9.732	-9.465
Quercetin	-6.464	-6.909	-8.348	-8.753
Curcumin	-6.558	-7.299	-7.567	-7.955
Resveratrol	-6.657	-7.021	-6.877	-7.52
Budesonide	-5.533	-7.024	-6.849	-7.677
Trimebutine	-6.748	-5.977	-7.384	-6.08
Colchicine	-5.601	-6.085	-6.697	-7.427
Fraxinellone	-5.229	-6.136	-6.638	-6.337
Pirfenidone	-5.131	-5.996	-6.35	-6.888
Metformin	-5.268	-4.991	-5.133	-4.909
Aminocaproic acid	-4.572	-4.444	/	/

**Figure 11:**
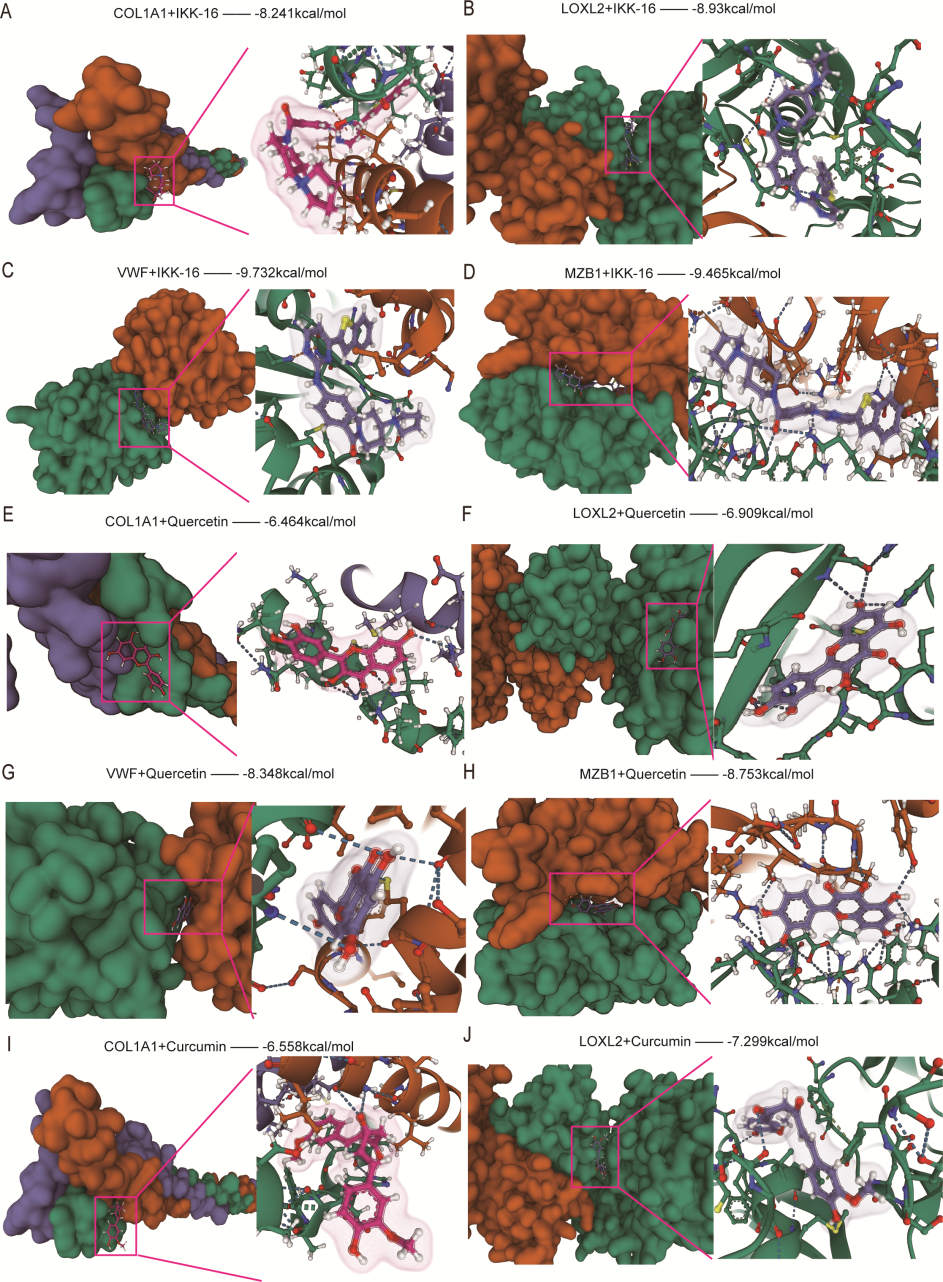
Molecular docking analysis of small molecules with fibrosis-associated proteins. **(A–D)** Binding modes of the small molecule IKK-16 with fibrosis-associated proteins. **(E–H)** Binding modes of the small molecule Quercetin with fibrosis-associated proteins. **(I, J)** Binding modes of the small molecule Curcumin with COL1A1 and LOXL2.The left panels display the overall three-dimensional structures of the proteins and small molecules in the docking complex, where proteins are shown as molecular surfaces and small molecules are shown in stick representation. The right panels magnify the docking sites, showing the specific interactions between the proteins and small molecules (e.g., hydrogen bonding and hydrophobic interactions). Color scheme: green for β-sheets, orange for α-helices, and purple for random coils. Magenta boxes highlight the small molecules, and dashed lines represent hydrogen bonds or other interactions. The binding energy (in kcal/mol) indicates docking stability, with lower values representing higher stability.

**Figure 12:**
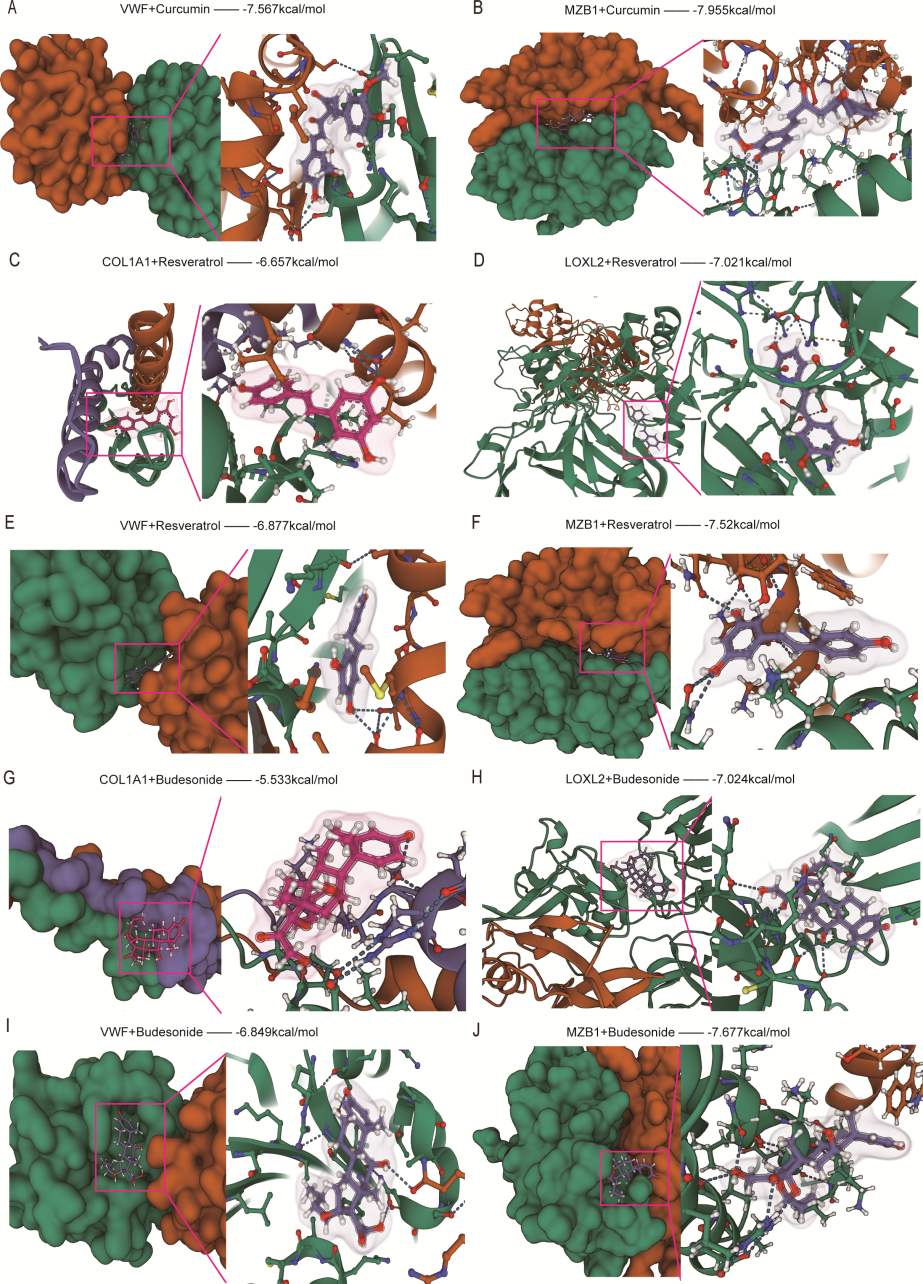
Molecular docking analysis of small molecules with target proteins. **(A, B)** Binding modes of the small molecule Curcumin with VWF and MZB1. **(C–F)** Binding modes of the small molecule Resveratrol with target proteins. **(G–J)** Binding modes of the small molecule Budesonide with target proteins. The left panels show the overall docking sites, while the right panels zoom in on the interaction details. Color scheme: green for β-sheets, orange for α-helices, and purple for random coils. Magenta boxes indicate small molecules, and dashed lines represent hydrogen bonds or other interactions. Binding energy (kcal/mol) reflects docking stability, with lower values indicating stronger binding.

The results indicated that all candidate drugs bound to their protein targets through visible hydrogen bonds and strong electrostatic interactions. Among these, the small molecule compound IKK-16 exhibited the highest affinity for its target genes, with binding energies below -8.0 kcal/mol (**Figures 11A-D**). Other drugs with highly stable binding include Quercetin (**Figures 11E-H**), Curcumin (**Figures 11I, J**, **Figures 12A, B**), and Resveratrol (**Figures 12C-F**), all of which have binding energies below -6.0 kcal/mol. The remaining candidate drugs, such as Budesonide (**Figures 12G-J**), Trimebutine (S3A-D), Colchicine (S3E-H), Fraxinellone (S3I-J, S4A-B), and Pirfenidone (S4C-F), had binding energies lower than -5.0 kcal/mol, indicating stable binding. Metformin showed moderate binding with its target genes, with binding energies around -5.0 kcal/mol (S4G-J). The weakest binding was observed for Aminocaproic acid, with binding energies of -4.572 and -4.444 kcal/mol with COL1A1 and LOXL2, respectively, and it was not displayed as a candidate drug. In conclusion, except for Aminocaproic acid, these drugs all exhibited good interactions with their target genes. Among the candidate compounds, IKK-16 exhibited the strongest binding affinities with the four core target proteins, with binding energies of −9.732 kcal/mol for VWF, −9.465 kcal/mol for MZB1, −8.930 kcal/mol for LOXL2, and −8.241 kcal/mol for COL1A1. These values represent the best binding affinities among all tested compounds, indicating strong selectivity and potential target specificity. Notably, the binding energy with VWF (−9.732 kcal/mol) was the lowest among all docking results in this study, suggesting a particularly stable interaction between IKK-16 and VWF. Based on binding affinities, the final ranking of the candidate small-molecule drugs is as follows: IKK-16 > Quercetin > Curcumin > Resveratrol > Budesonide > Trimebutine > Colchicine > Fraxinellone > Pirfenidone > Metformin. These findings may provide a useful reference for clinical drug selection.

## 3 Discussion

To determine the key role of ERS in the development of UC and its direct relevance to immune responses, this study used bioinformatics techniques such as machine learning to identify four hub genes, namely COL1A1, LOXL2, VWF, and MZB1. Immune infiltration analysis was employed to characterize the abundance of immune cells in two groups of colon tissues. Correlation analysis was then conducted to determine the significant associations between intestinal immune cells and central DE-ERG, which were validated across three external datasets and animal experiments. This highlighted the diagnostic potential of these four ER stress-related hub genes at different stages of UC. To clarify the relationship between these ER stress-related hub genes and intestinal fibrosis, we further explored the correlation between the expression levels of hub genes and gene set activity through the construction of a PPI network and GSVA analysis, validating their potential critical role in fibrosis regulation.

Intestinal fibrosis refers to the excessive deposition of extracellular matrix (ECM) components by activated mesenchymal-derived cells in the intestinal wall. It is a common complication of inflammatory bowel disease (IBD) and often leads to intestinal stricture (37). Studies have shown that 3.2% to 11.2% of UC patients may develop colorectal strictures, which often suggest the possibility of infiltrative cancer (38). In contrast to Crohn’s disease (CD), fibrosis and scarring in UC are confined to the mucosa (39). Studies have shown that 3.2% to 11.2% of UC patients may develop colorectal strictures, which often suggest the possibility of infiltrative cancer (18). Surgical intervention cannot prevent the formation of fibrosis, and there are currently no available anti-fibrotic therapies (37).Therefore, research into intestinal fibrosis in UC is urgently needed.

On the other hand, a series of recent reports have shown that ERS is a pathogenic factor in various inflammatory diseases (13). The unfolded protein response (UPR) aimed at alleviating ER stress by activating specific intracellular signaling pathways, including inositol-requiring enzyme 1 (IRE1), protein kinase R-like endoplasmic reticulum kinase (PERK), and activating transcription factor 6 (ATF6) (16, 17). These signaling pathways not only help restore ER homeostasis but also exacerbate tissue damage by promoting inflammation, apoptosis, and the release of fibrosis-related factors (11). Research on pulmonary diseases has found that the pro-fibrotic effects of increased ER stress are a critical component of the pathogenesis of idiopathic pulmonary fibrosis (IPF) (8). Research has shown that ERS can participate in the progression of pulmonary fibrosis by promoting apoptosis of type II alveolar epithelial cells, regulating inflammatory responses, inducing epithelial-mesenchymal transition, myofibroblast differentiation, and M2 macrophage polarization (9).Therefore, targeting ER stress and UPR components may offer therapeutic benefits in treating pulmonary diseases. However, as a typical inflammatory disease, is there a correlation between immune-inflammatory dysregulation, ERS and intestinal fibrosis?

Therefore, we conducted research using bioinformatics methods, including machine learning, immune infiltration, PPI network analysis, GSVA, and small molecule prediction, to elucidate the potential key role of ER stress (ERS) in the progression of UC-related intestinal fibrosis. During the screening of ERS-related differentially expressed genes, we found a positive correlation between ERS and the inflammatory process of UC. Further PPI network construction revealed interactions between hub genes and fibrosis-related genes. GSVA analysis confirmed that fibrosis gene sets are more readily activated in UC, and hub genes are positively correlated with UC activity. Ultimately, we identified the potential key role of ERS in the progression of UC-related intestinal fibrosis.

In constructing the PPI network of hub genes and fibrosis-related genes using STRING, we selected several key genes involved in classical fibrosis pathways, such as TGF-β1, TGFBR1, TGFBR2, and SMAD2, which are directly involved in the TGF-β signaling pathway (40, 41). Other genes included COL3A1 and COL5A2 (collagen synthesis) (42), FN1 (fibronectin) (43), MMP2 and MMP9 (involved in ECM degradation) (44), FLNA (filamin A) (45), and MYH11 (46), which is involved in vascular remodeling. About hub genes, the von Willebrand factor (VWF), primarily expressed in endothelial cells, is an endothelial-specific gene that plays a crucial role in vascular stability. Research has reported increased release of VWF in the inflamed colonic tissue of patients with inflammatory bowel disease (IBD) and chronic mouse colitis models, with VWF levels being higher during active phases compared to remission (47, 48). This elevation may contribute to the symptom of rectal bleeding observed in UC. MZB1, also known as pERp1 or MEDA-7, is an endoplasmic reticulum protein induced by plasma cells and is essential for antibody secretion and cell adhesion. It is a critical factor in endoplasmic reticulum stress (ERS) (49). Research indicate that MZB1-deficient mice are more susceptible to DSS-induced colitis (50). Although some studies have reported that MZB1 is upregulated in patients with pulmonary (51) and cutaneous (52) fibrosis, potentially related to B cell–mediated autoimmune responses (53), direct and robust functional evidence supporting its role in the pathogenesis of fibrosis remains limited. In addition, our PPI network analysis revealed that MZB1 exhibits weak associations with classical fibrosis-related genes, suggesting that it may not act as a direct driver of fibrogenesis. Future studies should focus on the potential bridging role of MZB1 between endoplasmic reticulum stress (ERS) and fibrosis, and evaluate its feasibility and safety as a therapeutic target. The human COL1A1 gene, located on chromosome 17q21, encodes the α1 chain of collagen and is considered a major gene involved in fibrosis. Research indicates that daily oral administration of the anti-fibrotic drug GED decreases COL1A1 expression and improves intestinal fibrosis in DSS-induced chronic colitis in mice (54). LOXL2 (Lysyl oxidase-like 2) is a Protein Coding gene, can crosslinks elastin and collagen, Circulating LOXL2 levels may be a noninvasive measure of intestinal fibrosis and GALT CD4^+^T lymphocyte depletion in treated HIV infection (55). These genes are involved in the onset and progression of fibrosis through mechanisms such as TGF-β receptor signaling, Smad signaling pathway, ECM component synthesis, and transcription factor regulation.

As the first anti-TNFα drug developed for inflammatory bowel disease (IBD), infliximab (IFX) effectively alleviates disease symptoms (56). However, 10-30% of IBD patients exhibit primary non-response (PNR), while 23-46% lose responsiveness over time (secondary non-response) (57, 58). To understand the molecular mechanisms underlying the efficacy and variability of clinical drug treatments, we further explored the responsiveness of hub genes to commonly used drugs, such as infliximab (IFX) and golimumab (GLM). The results indicated that LOXL2 does not respond to GLM, likely due to differences in the drug’s own potency and immune regulatory effects. Reports suggest that treatment failure with infliximab is associated with subclinical fibrosis in Crohn’s disease (59). Therefore, developing a drug specifically for the treatment of UC-related intestinal fibrosis is urgently needed.

Ulcerative colitis (UC) is a recurrent inflammatory disease, where each flare-up worsens intestinal inflammation, leading to increased extracellular matrix deposition and intestinal tissue damage. Repeated flare-ups over time enhance the degree of fibrosis during tissue repair. Therefore, when predicting potential drugs, we not only considered the docking results of fibrosis-related genes, such as COL1A1 and LOXL2, but also the binding affinities of immune-related gene MZB1 and hemostatic key gene VWF. Recent preclinical studies indicate that certain therapeutic drugs, particularly pirfenidone, exhibit beneficial effects in DSS-induced colitis (37). Furthermore, we listed several drugs that may treat intestinal fibrosis (**Table 1**) and performed molecular docking on the top 10 ranked drugs. Interestingly, during the docking process, we observed that small molecules containing more aromatic rings and rotatable bonds exhibited stronger binding abilities to target proteins. The multi-component molecular docking results provide robust data support for the selection of drugs to treat UC-related intestinal fibrosis in clinical practice. The selected drugs not only alleviate intestinal fibrosis but also participate in anti-inflammatory and hemostatic processes. Among all candidate compounds, IKK-16 exhibited the highest binding affinity toward VWF (−9.732 kcal/mol), suggesting strong and stable binding potential. It also demonstrated favorable binding energies with MZB1 (−9.465 kcal/mol), LOXL2 (−8.930 kcal/mol), and COL1A1 (−8.241 kcal/mol), outperforming other compounds and indicating potential selectivity or target preference. Despite its top-ranking docking performance, the biological relevance of IKK-16 to UC or intestinal fibrosis remains to be fully elucidated. Previous studies have shown that IKK-16, as an IκB kinase (IKK) inhibitor targeting the NF-κB signaling pathway, can alleviate TNF-α–induced epithelial inflammation in models of PBLD deficiency, thereby restoring intestinal barrier integrity (60). Given the critical role of NF-κB signaling in both inflammation and fibrosis, IKK-16 may hold therapeutic promise for UC-associated fibrotic processes. However, further experimental validation is warranted to confirm its efficacy in this specific context. Additionally, through network pharmacology, we unexpectedly discovered that several commonly used natural plant medicines for UC treatment, such as Coptis chinensis, Sophora japonica, Imperata cylindrica, and Hedyotis diffusa, contain quercetin, while Rheum officinale contains resveratrol, and Pulsatilla chinensis contains colchicine. These compounds have been verified in this study through molecular docking, demonstrating stable affinity with fibrosis-related target genes, and could serve as a reference for clinical selection of plant-based drugs to alleviate UC-related intestinal fibrosis progression.

Previous research has explored the relationship between ERS and UC (61, 62), yet our study employs a larger sample size and dataset, complemented by animal experiment validations. Our study not only elucidates the molecular mechanisms of ERS in the pathogenesis of ulcerative colitis (UC) but also provides preliminary evidence for the potential role of ERS in UC-associated intestinal fibrosis. However, this study has several limitations. First, gene validation was restricted to qPCR in animal models, without functional experiments to confirm the causal roles of key genes in ER stress-related fibrosis. Second, although IKK-16 showed promising docking affinity with four central proteins, its efficacy, specificity, and safety remain unvalidated, and the risk of off-target effects is notable given the structural diversity of the targets. Third, the molecular docking analysis was based on single-run predictions using AutoDock Vina, lacking cross-validation and failing to account for protein dynamics and pharmacokinetic factors, which limits the reliability of the results. Future studies should incorporate functional assays such as gene knockdown or overexpression, systematically evaluate the pharmacological properties of IKK-16, and adopt more robust docking strategies combined with *in vitro* and *in vivo* validation to better assess the therapeutic potential of candidate compounds.

## 4 Conclusion

In conclusion, we identified a close association between COL1A1, LOXL2, VWF, MZB1, and ERS in the pathogenesis of ulcerative colitis (UC). Among these, COL1A1, LOXL2, and VWF, as fibrosis-related genes, may serve as key mechanisms and potential targets for ERS-induced UC intestinal fibrosis. The small molecule IKK-16 could be a potential key drug for the treatment of UC-related intestinal fibrosis.

## 5 Materials and methods

### 5.1 Datasets acquisition and pre-processing

We searched the GEO database using the keyword “ulcerative colitis” and selected datasets according to the following criteria: (1) inclusion of human colon tissue samples from both UC and control groups; (2) transcriptome-wide expression data (microarray or RNA-seq); (3) publicly available datasets; and (4) a minimum of 5 samples per group. Based on these criteria and the study objectives, six mRNA expression datasets (GSE206285, GSE92415, GSE87466, GSE66407, GSE128682 and GSE73661) were ultimately included for integrative analysis. **Table 3** lists the basic details of these datasets. Sets of 2,164 endoplasmic reticulum stress-associated genes were obtained from the Gene Cards database (https://www.genecards.org/) with scores > 5. Additionally, 1,323 genes with a score ≥ 9 were selected from the Gene Cards database as the fibrosis-associated gene set (FGS).

**Table 3:** The information of all the datasets in the study.

GEO ID	Platform	RNA type	Used sample	Sample source
GSE206285	GPL13158	mRNA	CON: UC = 18:550	Colonic mucosal
GSE92415	GPL13158	mRNA	CON: UC = 21:162	Colonic mucosal
GSE36807	GPL570	mRNA	CON: UC = 7:15	Colonic mucosal
GSE66407	GPL19833	mRNA	non-inflammatory: inflammatory = 94:62	Colonic mucosal
GSE128682	GPL21697	mRNA	active: remission = 14:14	Colonic mucosal
GSE73661	GPL6244	mRNA	Before: After (IFX) = 23:23	Colonic mucosal

### 5.2 Differentially expressed genes identification and function and pathway enrichment analysis

GEO2R (https://www.ncbi.nlm.nih.gov/geo/geo2r/) was used to obtain the genes expressed differently with Adjusted P-value < 0.05 and a | log2 (fold change) | ≥ 1 were considered to be statistically significant. Volcano plots of the results were drawn through http://www.xiantao.love, a comprehensive web service for biomedical data analysis and visualization. Kyoto Encyclopedia of Genes and Genomes (KEGG) pathway enrichment analysis, and Reactome pathway enrichment analysis was performed using the OmicShare tools, a free online platform for data analysis (https://www.omicshare.com/tools). FDR < 0.05 was considered significant.

### 5.3 Machine learning algorithms for recognition of hubs

In this study, we use 2 machine learning algorithms: Least Absolute Shrinkage and Selection Operator (LASSO) regression and Random Forest (RF), to screen Hubs of Differentially ERS-related genes for UC (63, 64). We first performed data preprocessing, which included normalization and missing value imputation. Subsequently, we conducted LASSO regression analysis using the R package “glmnet” and applied 10-fold cross-validation to select ERS-related differentially expressed genes (DEGs) for inclusion in the predictive model. We then constructed a random forest using the R package “randomForest,” employing two feature importance assessment methods: Incremental Mean Squared Error (IncMSE) and Gini coefficient (IncNodePurity), to enhance the model’s interpretability and robustness. Finally, receiver operating characteristic (ROC) analysis was performed using the “pROC” package to assess the efficacy of hubs to diagnose UC.

### 5.4 Assessment of immune infiltration patterns in UC

In order to analyze the Spearman association between unique diagnostic markers and immune invading cells, immune cell infiltration analysis was carried out using the CIBERSORT (0.1.0) package [25822800], which can predict the immune cell composition of tissues using the Cibersort deconvolution algorithm based on input gene expression profiles and built-in reference set LM22. Permutation (PERM) was established to 1000 for more stable results. Differential analysis and visualization of immune cells between groups were presented using the “ggboxplot” package. The correlation analysis and visualization between hubs and between hubs and immune cells was completed through Xiantao.

### 5.5 Protein-protein interaction

To identify key protein-protein interactions (PPI) and associated biological pathways, we constructed a PPI network for fibrosis-related genes using the STRING database (version 11.5; https://string-db.org/). The input gene list, including COL1A1, LOXL2, MZB1, and VWF, was derived from differentially expressed gene (DEG) analysis and classical fibrosis pathway genes obtained through literature review. A minimum interaction confidence score of 0.4 (medium confidence) was applied, and disconnected nodes were excluded. Visualization and network analysis were performed using Cytoscape to highlight key hub genes. Functional enrichment analysis was conducted using the Reactome database, Gene Ontology and WikiPathways to identify enriched pathways, with a focus on extracellular matrix (ECM) organization and related processes. The enrichment results were visualized as dot plots, showing the FDR values, gene counts, and signal strength for each pathway.

### 5.6 Gene set variation analysis

GSVA (Gene Set Variation Analysis) is a method used to evaluate the activity of gene sets within samples, particularly suitable for the analysis of gene expression data. GSVA was utilized to calculate pathway enrichment scores for fibrosis-related gene sets in two GEO datasets (GSE206285 and GSE92415). The fibrosis-related gene set was curated from the Gene Card databases, representing genes known to be involved in fibrotic processes. Normalized gene expression matrices were input into the GSVA R package (version 3.6), and the analysis was performed using the non-parametric kernel estimation method to generate GSVA scores for each sample. The enrichment scores were compared between ulcerative colitis (UC) and control (CON) groups using violin plots to visualize the distribution of pathway activity. Spearman correlation analysis was conducted to assess the relationship between GSVA scores and the expression levels of COL1A1, LOXL2, VWF, and MZB1 in the UC datasets. The analyses were performed using R software (version 3.6), with visualization carried out using the ggplot2 package. Statistical significance was determined with a two-tailed P-value < 0.05.

### 5.7 Drug screening and molecular docking analysis

We performed drug screening using three approaches. First, the Connectivity Map (CMap, https://clue.io/) database was utilized to predict potential compounds based on differential gene expression analysis. The compound with the top-ranked TAG score was selected for subsequent molecular docking (**Supplementary Table 1**). Second, a literature search was conducted on PubMed using the query “(intestinal fibrosis [Title/Abstract]) AND (drug [Title/Abstract])” to identify drugs reported for the treatment of intestinal fibrosis. Additionally, the DrugBank database was searched using the term “intestinal fibrosis” to collect potential therapeutic drugs. The chemical structures of candidate drugs/small molecules were obtained from the PubChem compound database (https://pubchem.ncbi.nlm.nih.gov/), with specific compound IDs listed in **Supplementary Table 2**.The primary protein structures of target genes, including COL1A1 (PDB ID: 5CTD; resolution: 1.6 Å), LOXL2 (PDB ID: 5ZE3; resolution: 2.4 Å), VWF (PDB ID: 1AO3; resolution: 2.2 Å), and MZB1 (PDB ID: 7AAH; resolution: 1.4 Å), were downloaded from the Protein Data Bank (http://www.rcsb.org, PDB).

Molecular docking was performed using AutoDock Vina 1.2.2 (http://autodock.scripps.edu/), a widely used computational software for protein–ligand docking, to evaluate the binding affinities between the candidate compounds and the target proteins (VWF, MZB1, COL1A1, and LOXL2) (65). All protein structures were preprocessed by removing water molecules and adding polar hydrogen atoms, followed by conversion into the PDBQT format. Binding pockets for each target were defined based on their structural characteristics. For proteins with co-crystallized ligands, the docking grid box was centered on the ligand-binding site. For proteins lacking ligand information, potential binding cavities were identified using the cavity detection module in PyMOL, and further refined based on conserved functional domain annotations from the UniProt database. The grid box was centered to encompass each protein’s domain, allowing for free molecular movement, and the interface pocket was set as a cubic box of 30 Å × 30 Å × 30 Å with a grid spacing of 0.5 Å. Finally, the docking models were visualized using PyMOL (https://pymol.org/).

### 5.8 DSS-induced UC mice model

Male C57BL/6J mice (24–26 g) were obtained from GemPharmatech (Chengdu) Co., Ltd. [Animal License Number: SCXK (Chuan) 2020-034]. This study was conducted in accordance with the recommendations in the Guide for the Care and Use of Laboratory Animals published by the National Institutes of Health. The protocol was approved by the Committee on the Ethics of Animal Experiments of Chengdu University of Traditional Chinese medicine, [Approval Number: 2024004]. The animals were randomly fed standard rodent food and water at ambient with temperature (23 ± 1°C) and subjected to a 12-hour light/dark cycle in a pathogen-free pen. Randomly divide the mice into a control group and a DSS group. Provide the DSS group mice with drinking water containing 3% dextran sodium sulfate (DSS) for 7 days, while the control group was only given pure water. Evaluate daily body weight, fecal consistency, and fecal bleeding (66).

### 5.9 Hematoxylin and eosin staining

In the normal and UC groups, colon tissue was fixed in 4% paraformaldehyde buffer for 2 days. After paraffin embedding, the samples were sliced with a thickness of 5mm and stained with HE for histopathological examination using an optical microscope (Olympus Corporation, Tokyo, Japan).

### 5.10 Enzyme-linked immunosorbent assay

After successful model establishment, mice in each group were mildly anesthetized with isoflurane and whole blood was collected via retro-orbital bleeding into centrifuge tubes. The samples were left undisturbed at room temperature for 30 minutes to allow clotting, followed by centrifugation at 3000 rpm for 10 minutes at 4°C. The supernatants (serum) were carefully transferred into RNase-free tubes and stored at –80°C until ELISA analysis. According to the manufacturer’s instructions, ELISA kits, ELISA kits (Elabscience, Wuhan, China) were used to detect tumor necrosis factor (TNF)-α, interleukin- 6(IL- 6), Interleukin-1β (IL-1β), and interferon (IFN)-γ levels in the supernatant. Then, the absorption coefficients were applied to calculate TNF-α, IL-6, IL-1β and IFN-γ concentrations.

### 5.11 RNA extraction and RT-qPCR

Animal Total RNA Isolation Kit (World’s Foregene, RE-03011/03014) was used to isolate total mRNA from colon tissues, and cDNA was prepared using 2× RT OR-Easy TM Mix (World’s Foregene, RT-01021/01022/01023), according to the manufacturer’s instructions. We then analyzed the resulting cDNA by RT-qPCR using the Real Time PCR Easy TM-SYBR Green I (No Rox, World’s Foregene, QP01011/01012/01013/01014). The primer sequences are seen in **Table 4**. Target mRNA expression levels were analyzed using the comparative 2-Ct method by normalizing to levels of β-actin.

**Table 4:** The results of primer design.

Primer Name	Sequence	Product Length	ID
Vwf-1F	CTTCTGTACGCCTCAGCTATG	125	22371
Vwf-1R	GCCGTTGTAATTCCCACACAAG		
Mzb1-1F	CCACTGTTGCTACTGTTAGGG	200	69816
Mzb1-1R	GTGTGAGATTTAGCCTCTGCTT		
Col1a1-1F	GCTCCTCTTAGGGGCCACT	91	12842
Col1a1-1R	ATTGGGGACCCTTAGGCCAT		
Loxl2-1F	CGGAAAGCCTACAAGCCAGA	123	94352
Loxl2-1R	GTCCCACTTGTCATCGCAGA		

### 5.12 Statistical analysis

SPSS 26.0 software was used for statistical analysis, and the measurement data were expressed as Mean ± SD. Perform normality tests and tests for homogeneity of variance prior to statistical analysis. Normality was tested with Shapiro-Wilk test. For comparisons between two independent sample groups, if the data met the criteria for normal distribution and homogeneity of variance, we used a two-independent-samples t-test. If the data were normally distributed but had unequal variances, we employed the more robust Welch test. If the data did not meet the criteria for normal distribution and homogeneity of variance, the non-parametric two-tailed Mann-Whitney U-test was used. Mouse body weight data were statistically analyzed using repeated measures analysis of variance and Bonferroni method. P<0.05 indicates statistically significant difference. Pictures were drawn with GraphPad Prism software (version 8.0, GraphPad Prism Software Inc, CA, United States).

## Data Availability

The datasets analyzed in this study are publicly available from the GEO database (https://www.ncbi.nlm.nih.gov/geo/) under accession numbers GSE206285, GSE92415, GSE87466, GSE66407, GSE128682 and GSE73661. The experimental validation data (e.g., qPCR, ELISA) supporting the conclusions of this article are available from the corresponding author upon reasonable request.
