# Supplementary material for: ER stress genes (COL1A1, LOXL2, VWF) predicts IKK-16 as a Candidate therapeutic target for colitis-related inflammation and fibrosis suppression
Source: Front Immunol. 2025 Jun 18;16:1587860. doi: 10.3389/fimmu.2025.1587860 (PMC12213447; doi:10.3389/fimmu.2025.1587860)

Supplementary Material

# Supplementary Figures and Tables

## Supplementary Figures


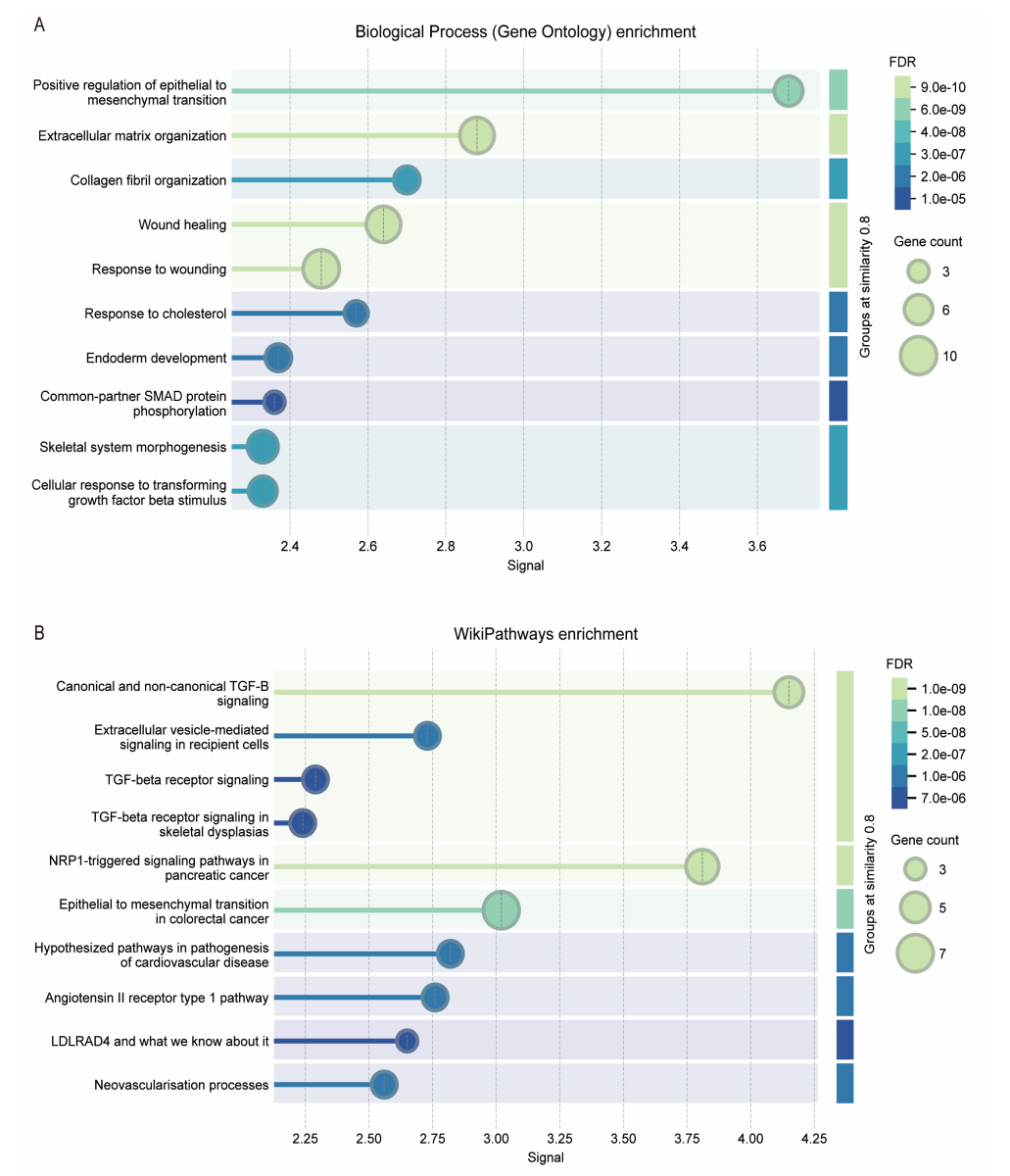


**Supplementary Figure 1.Pathway enrichment analysis of hub genes and classical fibrosis-related genes. (A)** Gene Ontology (GO) biological process enrichment analysis. **(B)** WikiPathways enrichment analysis. The top enriched terms are visualized, with dot size representing the gene count per term and color intensity indicating the FDR value.


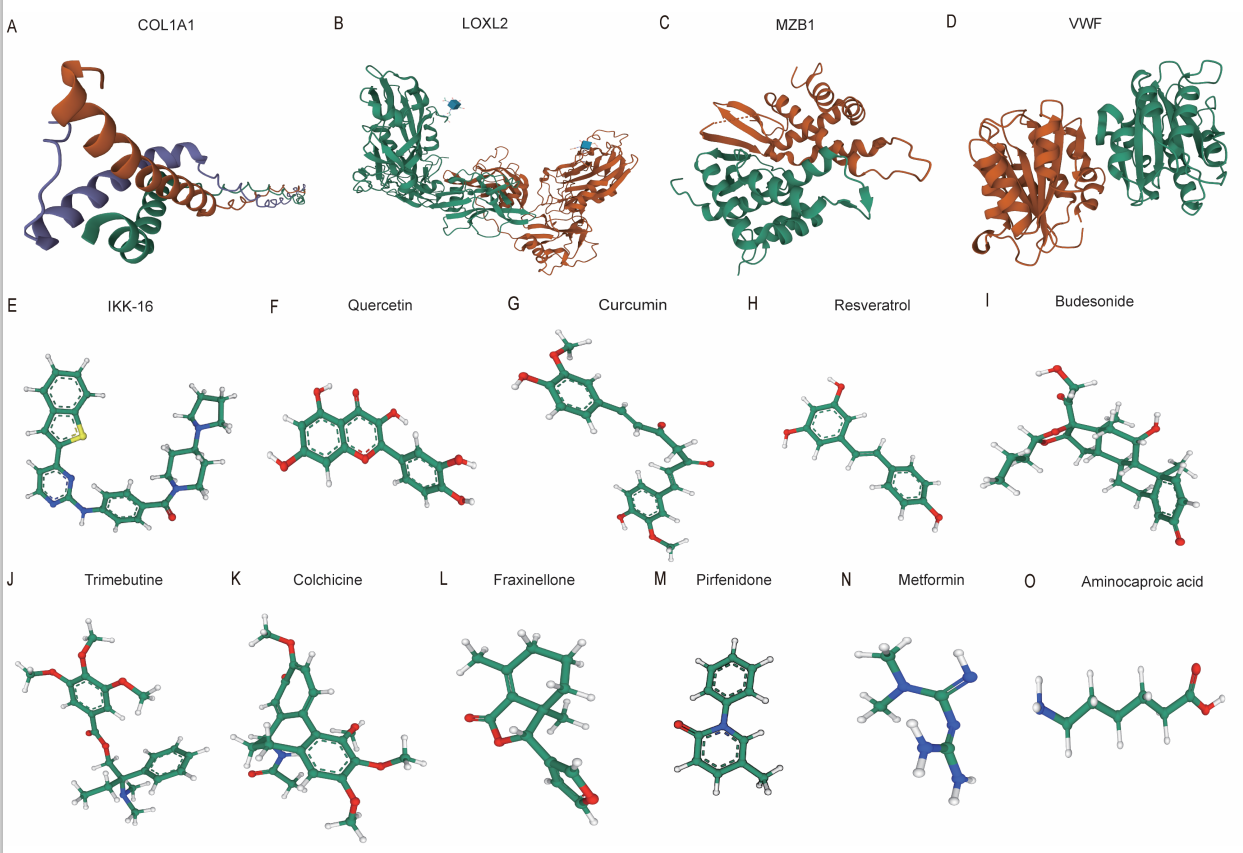


**Supplementary Figure 2.Protein structures related to intestinal fibrosis and potential small-molecule compounds.(A–D)** Three-dimensional structures of key proteins associated with intestinal fibrosis progression: COL1A1 **(A)**, LOXL2 **(B)**, MZB1 **(C)**, and VWF **(D)**.**(E–O)** Three-dimensional structures of potential antifibrotic small-molecule compounds identified through computational screening and literature review: IKK-16 **(E)**, Quercetin **(F)**, Curcumin **(G)**, Resveratrol **(H)**, Budesonide **(I)**, Trimebutine **(J)**, Colchicine **(K)**, Fraxinellone **(L)**, Pirfenidone **(M)**, Metformin **(N)**, and Aminocaproic Acid **(O)**.Protein structures are represented in colored ribbon diagrams, with different colors indicating secondary structures to reflect conformational differences in protein functional domains. Small molecules are shown as three-dimensional optimized molecular structures, where green represents carbon (C) atoms, red represents oxygen (O), blue represents nitrogen (N), yellow represents sulfur (S), and white represents hydrogen (H).


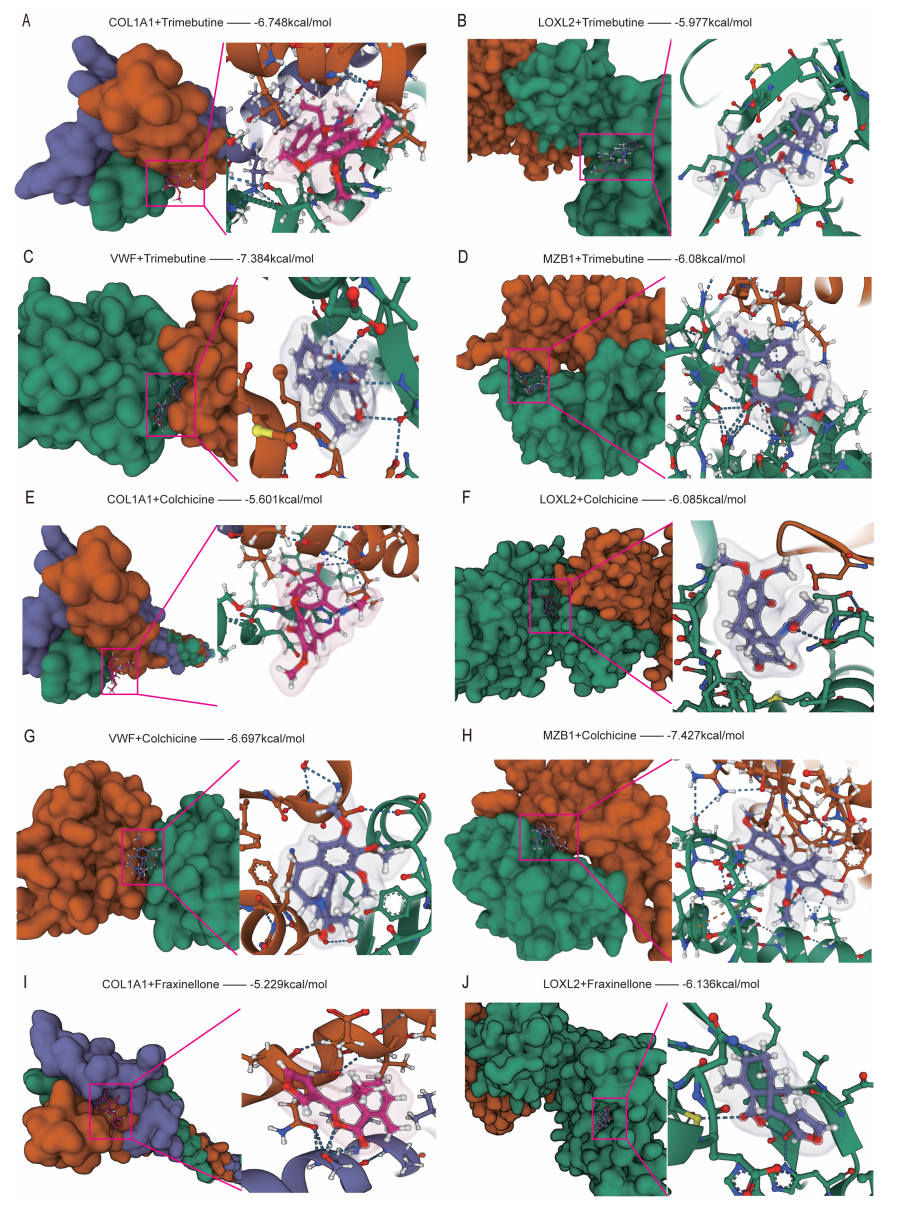


**Supplementary Figure 3. Molecular docking analysis of small molecules with target proteins.(A–D)** Molecular docking results of Trimebutine with target proteins.**(E–H)** Molecular docking results of the small molecule Colchicine with target proteins.**(I–J)** Molecular docking results of Fraxinellone with COL1A1 and LOXL2.The left panels show the overall binding sites, while the right panels zoom in on detailed views of the docking sites. Color scheme: green for β-sheets, orange for α-helices, and purple for random coils. Magenta boxes highlight the small molecules, and dashed lines represent hydrogen bonds or other interactions. The binding energy (in kcal/mol) indicates docking stability, with lower values representing stronger binding.


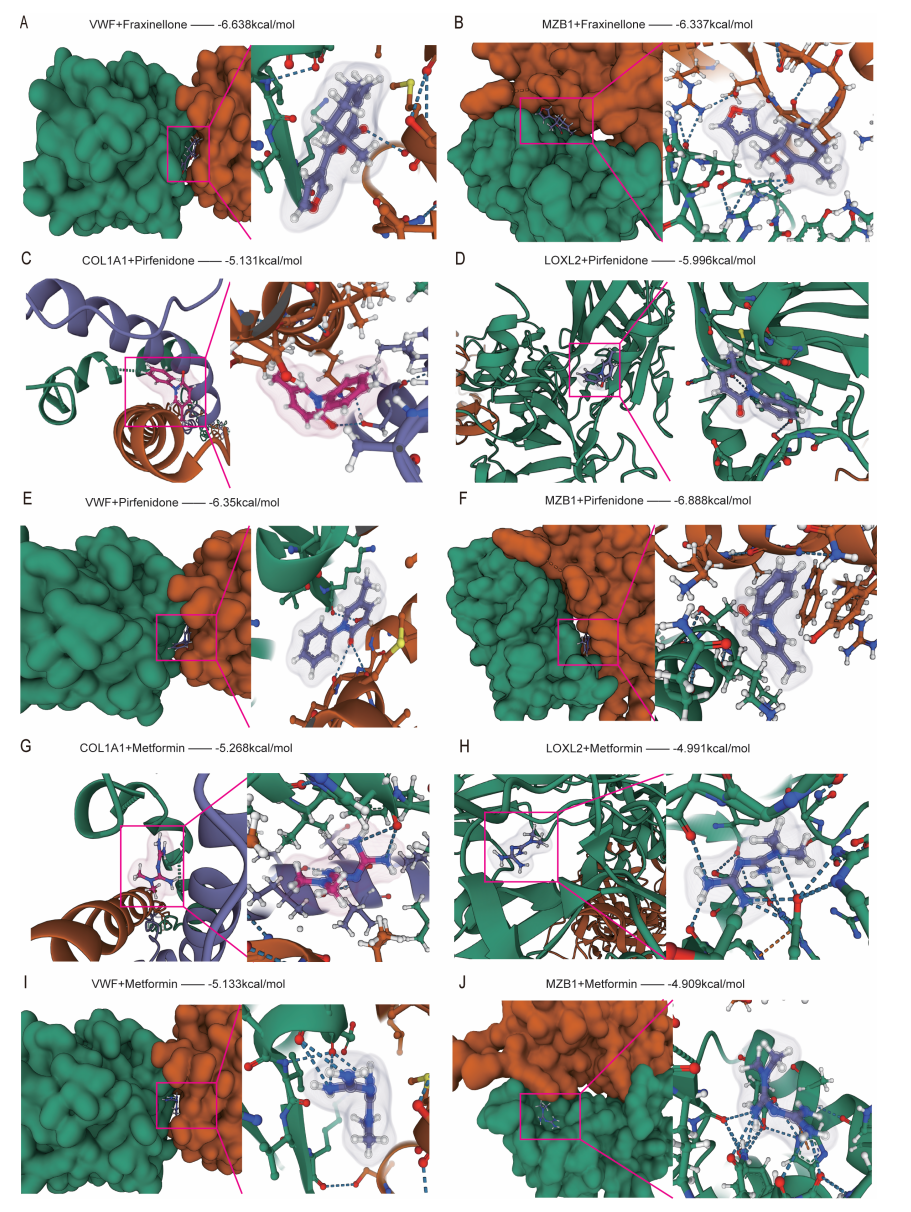


**Supplementary Figure 4. Molecular docking analysis of target proteins with additional small molecules.(A–B)** Molecular docking results of the small molecule Fraxinellone with VWF and MZB1.**(C–F)** Molecular docking results of Pirfenidone with target proteins.**(G–J)** Molecular docking results of Metformin with target proteins.The left panels show the overall binding sites, while the right panels zoom in on detailed views of the docking sites.Color scheme: green for β-sheets, orange for α-helices, and purple for random coils. Magenta boxes highlight the small molecules, and dashed lines represent hydrogen bonds or other interactions. The binding energy (in kcal/mol) reflects docking stability, with lower values indicating stronger binding.


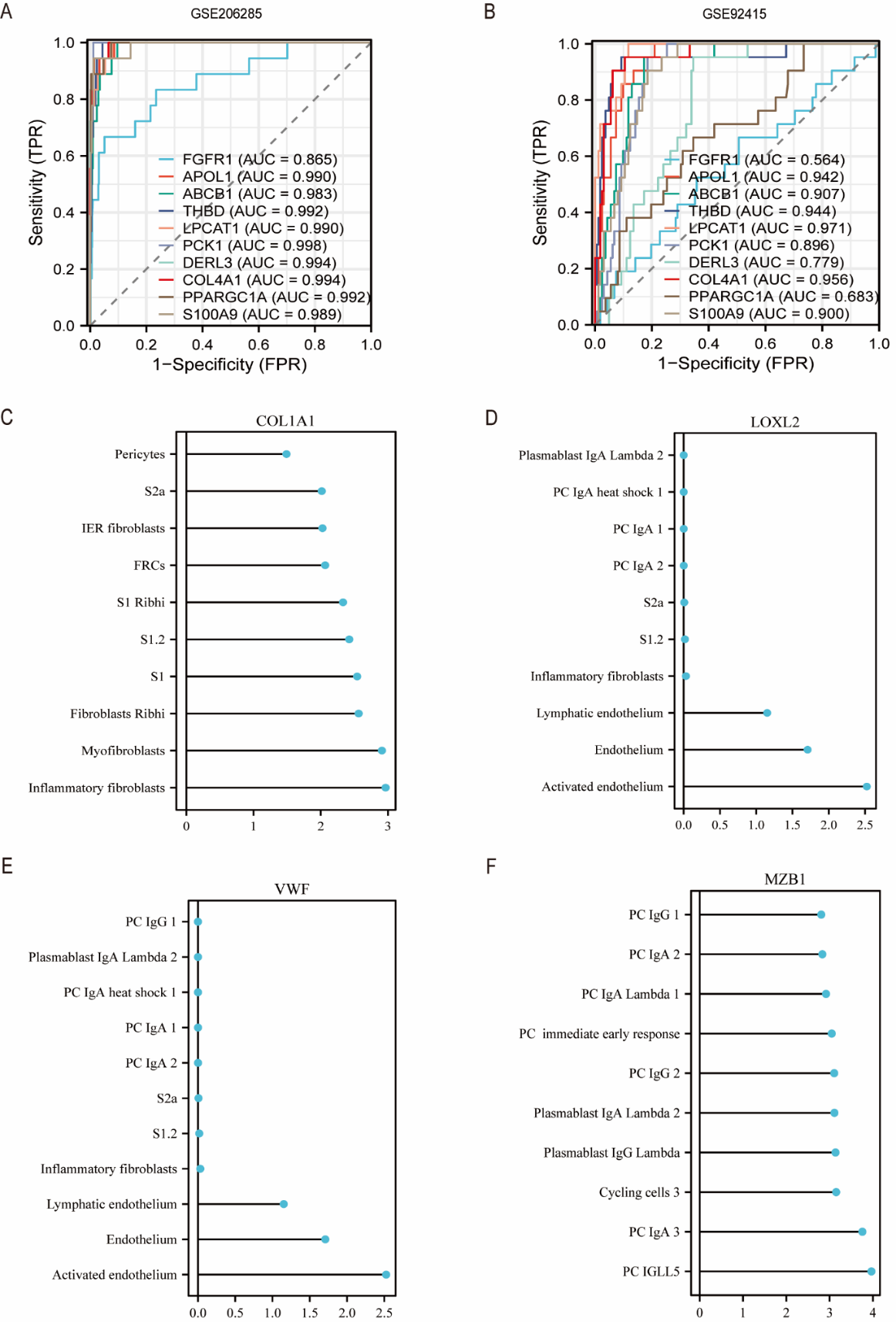


**Supplementary Figure 5. Supplementary ROC curve plots and single-cell analysis figures. (A-B)** ROC curves of machine learning-identified genes (excluding the hub genes) from Figure G in the GSE206285**(A)** and GSE92415**(B)** datasets. **(C–F)** Bar plots show the expression levels of four key genes—COL1A1 **(C)**, LOXL2 **(D),** VWF **(E)**, and MZB1 **(F)**—across different cell types as identified by single-cell RNA sequencing analysis. The Y-axis indicates the annotated cell types, and the X-axis represents the relative expression level of the corresponding gene. Dot and line elements represent the average expression and distribution within each cell type, respectively.


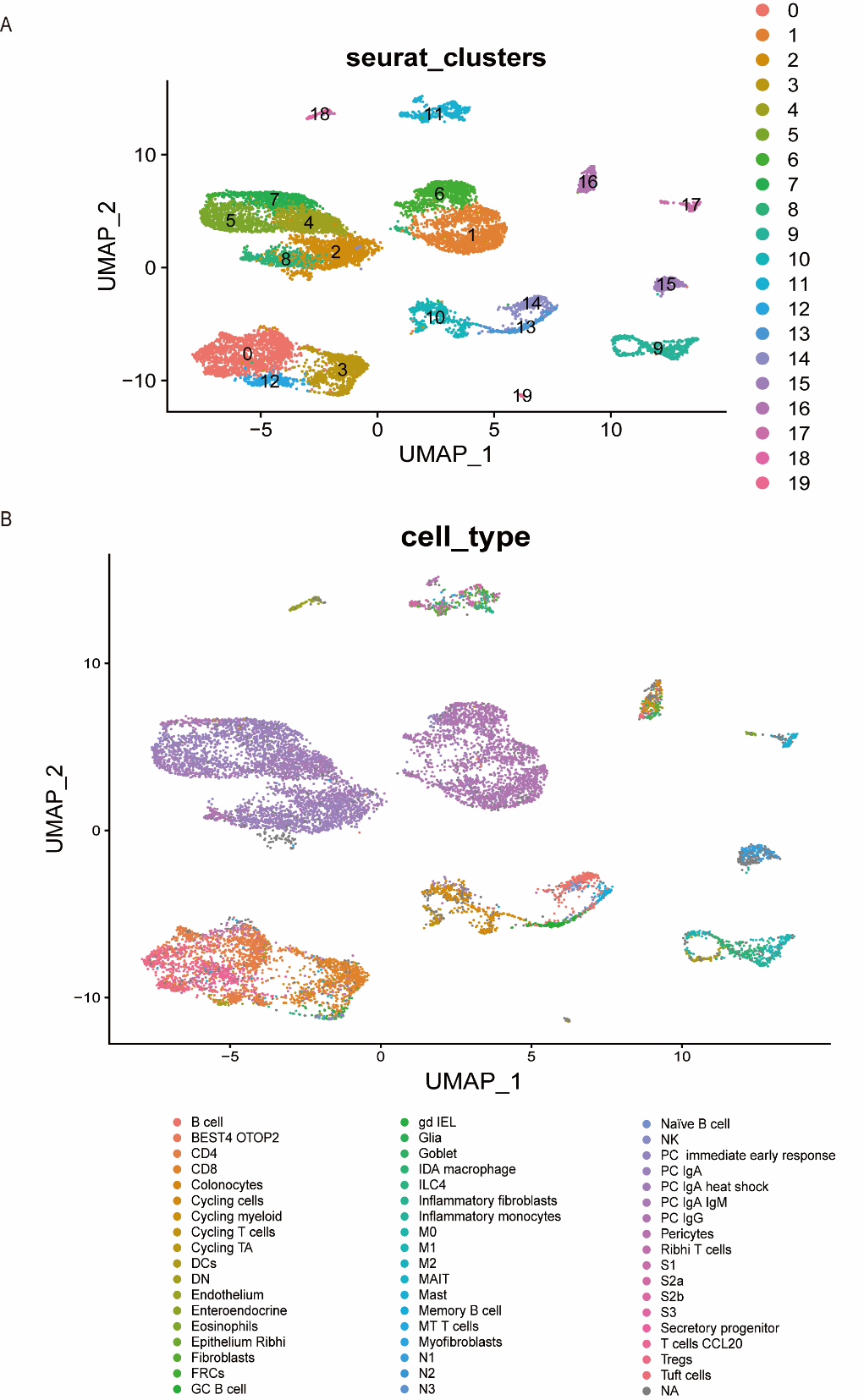


**Supplementary Figure 6. UMAP visualization of single-cell transcriptomes. (A)** Clustering of cells using Seurat-based unsupervised clustering algorithm. Each cluster is labeled by a numeric identifier (0–19) and distinguished by a unique color. **(B)** Cell type annotation of clusters based on marker gene expression profiles. Each dot represents a single cell, colored by its assigned cell type. A total of **54** cell types were identified, including immune cells (e.g., B cells, T cells, monocytes), epithelial subtypes (e.g., BEST4+ cells, goblet cells, enterocytes), stromal cells (e.g., fibroblasts, myofibroblasts), and others. The legend shows the corresponding color for each identified cell type.

## Supplementary Tables

**Supplementary Table 1** **CMAP small molecule prediction results with TAG less than -0.85.**

| **pert_id** | **pert_iname** | **moa** | **TAG** |
| --- | --- | --- | --- |
| BRD-K14618467 | IKK-16 | IKK inhibitor | -0.8938 |
| BRD-K49049886 | CGS-15943 | Adenosine receptor antagonist | -0.8883 |
| BRD-K89329876 | BRD-K89329876 | Kinesin inhibitor | -0.8731 |
| BRD-K21672174 | Ro-28-1675 | Glucokinase activator | -0.86 |
| BRD-K70241288 | L-692585 | Growth hormone releasing peptide ligand agonist | -0.8577 |
| BRD-A97730597 | hexylcaine | Sodium channel blocker | -0.8564 |
| BRD-K71879491 | tretinoin | Retinoid receptor agonist\|Retinoid receptor ligand | -0.8558 |
| BRD-K85402309 | dovitinib | EGFR inhibitor\|FGFR inhibitor\|FLT3 inhibitor\|PDGFR tyrosine kinase receptor inhibitor\|VEGFR inhibitor | -0.8533 |
| BRD-K53523901 | arctigenin | MEK inhibitor | -0.8512 |

**Supplementary Table 2** **Docked Compound ID**

| **Serial Number** | **Compound Name** | **PubChem CID** |
| --- | --- | --- |
| 1 | IKK-16 | 9549298 |
| 2 | Curcumin | 969516 |
| 3 | Quercetin | 5280343 |
| 4 | Resveratrol | 445154 |
| 5 | Trimebutine | 5573 |
| 6 | Budesonide | 5281004 |
| 7 | Colchicine | 6167 |
| 8 | Fraxinellone | 124039 |
| 9 | Pirfenidone | 40632 |
| 10 | Metformin | 4091 |
| 11 | Aminocaproic acid | 564 |

**Supplementary Table 3** **Complete list of differentially expressed genes from GSE206285 and GSE92415.**

| GSE206285 | | | |
| --- | --- | --- | --- |
| **gene** | **logFC** | **padj** | **sig** |
| CHI3L1 | 5.608156 | 5.74E-35 | up |
| S100A8 | 5.480676 | 1.49E-28 | up |
| SAA1 | 4.491598 | 9.21E-23 | up |
| MMP1 | 4.277627 | 1.42E-18 | up |
| KLK10 | 4.272525 | 4.44E-13 | up |
| MZB1 | 4.196904 | 9.20E-58 | up |
| IGFBP5 | 4.074646 | 7.00E-40 | up |
| CCL11 | 3.979397 | 5.05E-70 | up |
| IGH | 3.932037 | 2.33E-77 | up |
| MMP3 | 3.921093 | 6.42E-17 | up |
| CLDN8 | -5.95207 | 2.50E-68 | down |
| PCK1 | -4.99752 | 1.58E-27 | down |
| SLC26A2 | -4.71344 | 5.31E-32 | down |
| TRPM6 | -4.05164 | 1.55E-31 | down |
| UGT2A3 | -3.60834 | 5.65E-49 | down |
| AQP8 | -3.5719 | 2.01E-24 | down |
| HOXD10 | -3.54983 | 5.36E-60 | down |
| PNLIPRP2 | -3.42591 | 3.43E-62 | down |
| HMGCS2 | -3.41976 | 1.78E-14 | down |
| TPH1 | -3.35327 | 1.54E-32 | down |

| GSE92415 | | | |
| --- | --- | --- | --- |
| **gene** | **logFC** | **padj** | **sig** |
| SLC6A14 | 4.853446 | 2.82E-44 | up |
| DUOX2 | 4.672704 | 6.25E-31 | up |
| MMP3 | 4.642072 | 2.34E-17 | up |
| CHI3L1 | 4.593919 | 2.97E-14 | up |
| DEFB4A | 3.920182 | 2.10E-09 | up |
| S100A8 | 3.769731 | 1.69E-14 | up |
| REG3A | 3.623138 | 2.66E-09 | up |
| CXCL8 | 3.582969 | 3.96E-12 | up |
| SAA1 | 3.471637 | 6.22E-11 | up |
| CXCL1 | 3.43224 | 3.54E-20 | up |
| AQP8 | -4.40908 | 3.50E-17 | down |
| SLC51A | -3.71208 | 2.27E-26 | down |
| SLC26A2 | -3.4744 | 1.35E-11 | down |
| HMGCS2 | -3.4517 | 4.15E-22 | down |
| DPP10-AS1 | -3.42163 | 2.49E-36 | down |
| CLDN8 | -3.25356 | 6.67E-07 | down |
| SLC38A4 | -3.0834 | 1.31E-45 | down |
| PCK1 | -3.05595 | 1.97E-08 | down |
| MEP1B | -3.01804 | 6.51E-31 | down |
| GBA3 | -3.01253 | 1.42E-23 | down |

**Supplementary Table 4 Drug screening with complete inclusion criteria**


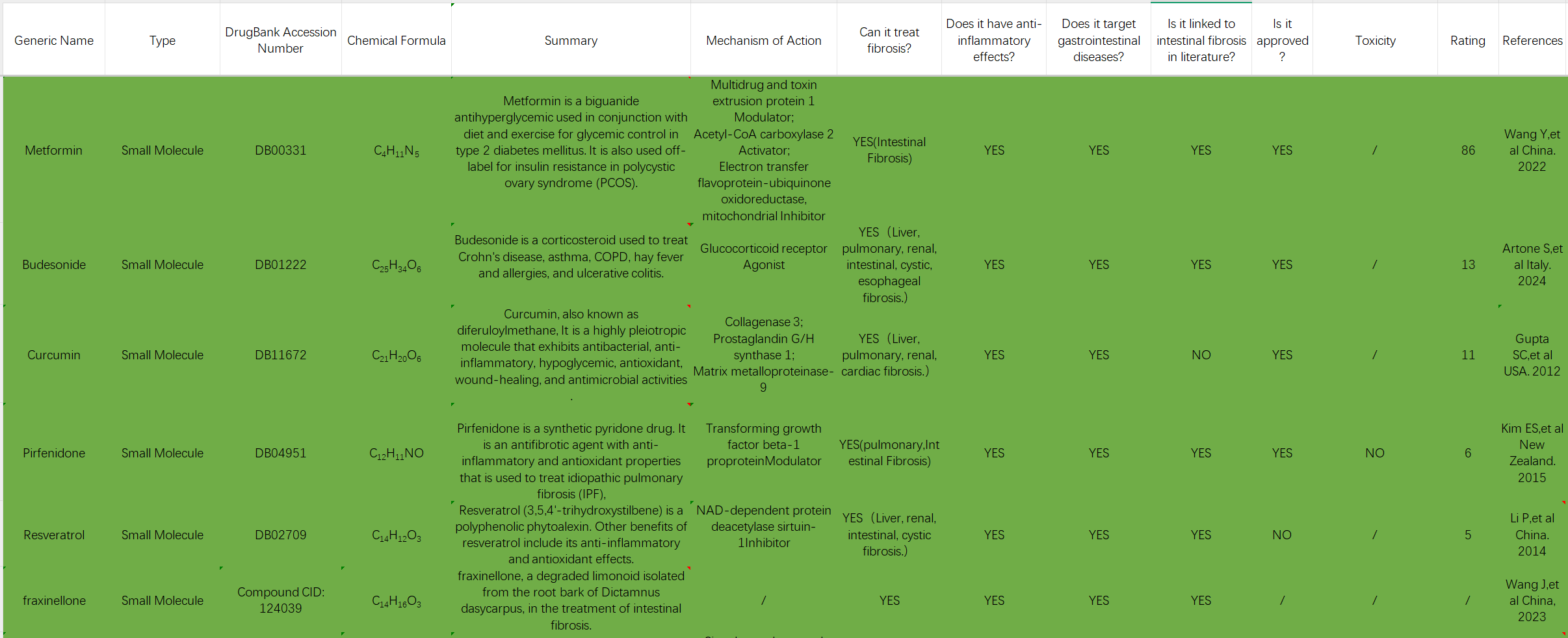


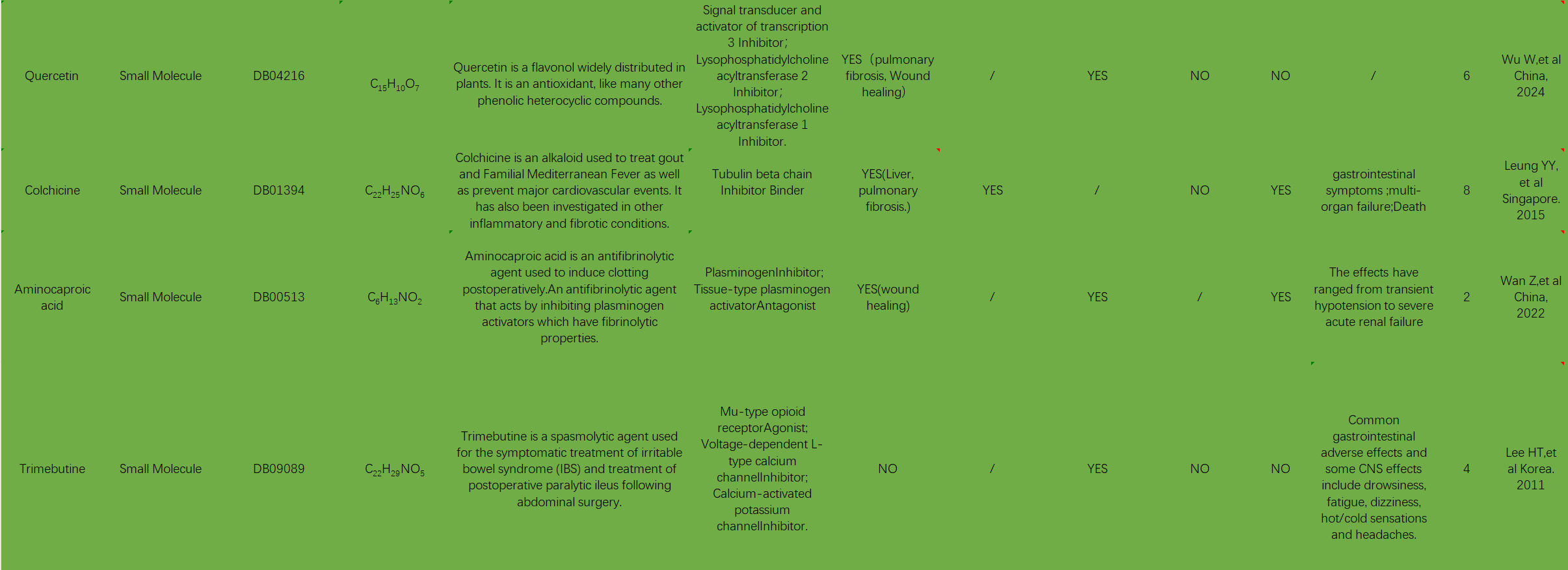

Supplement: Supplementary file 1 [file DataSheet1.docx]
